# Supplementary material for: Pre-processing visualization of hyperspectral fluorescent data with Spectrally Encoded Enhanced Representations
Source: Nat Commun. 2020 Feb 5;11:726. doi: 10.1038/s41467-020-14486-8 (PMC7002680; doi:10.1038/s41467-020-14486-8)
Supplement: Supplementary file 1 — Supplementary Information [file 41467_2020_14486_MOESM1_ESM.pdf]

Pre-processing visualization of hyperspectral fluorescent data with Spectrally Encoded Enhanced Representations

Shi, Koo et al

Supplementary Table 1: Parameters for in vivo imaging. All data points are 16 bit depth, acquired using LD C-Apochromat 40x/11 W lens.

	**Scaling X-Y [µm]**	**Scaling Z [µm]**	**Imaged Volume (x, y, z, λ, t) [pixels]**	**Pixel Dwell [µs]**	**Gain [au]**	**Pinhole [µm]**	**Beam Splitters**	**Laser power [%]**	**Size [GB]**
Fig1	1.661	15.837	x:2048, y: 768, z:20, channels:33	3.15 µs	825	73 µm	458	20 % @458 nm	1.93
Fig4, SupFig 13,14	0.208		x:1024, y:1024, channels:33	12.6 µs	820	601 µm	690+	1.8 % @740 nm	0.06
Fig5, Sup Movie 1, SupFig1 5, 16	0.923	6.000	x:3840, y:768, z:19, channels:33	3.15 µs	805	229 µm	488	5.5 % @488 nm	3.44
Fig6, Sup Movie 2, SupFig2 , 17, 18	0.415	5.000	x:2048, y:512, z:7, channels:33	6.30 µs	800	186 µm	458	1.0 % @458 nm	0.45
Fig7, SupFig1 9	0.104		x:2048, y:1024, channels:33	1.58 µs	827	40 µm	458	47.7 % @458 nm	0.13
SupFig 24, Sup Movie 3,4	0.069	0.069	x:1024, y:1024, channels: 33, time: 100	1.59 µs	800	70 µm	488/561	1.2% @ 561 nm 6.5% @ 488 nm	6.93
SupFig1	0.923	6	x:365, y:196, z:4, channels:33	6.30 µs	796	147 µm	458	5.8% @ 458 nm	0.02
SupFig1	0.052	1	x:451, y:825, z:6, channels:33	6.30 µs	827	40 µm	458 / 690+	60% @ 458 nm	0.14
SupFig1	0.052	1	x:1024, y:1024, z:6, channels:33	6.30 µs	827	40 µm	458 / 690+	60% @ 458 nm	0.39
SupFig1	0.865	2	x:512, y:512, z:70, channels:32	1.58 µs	827	601 µm	690+	12% @ 900 nm	1.10
SupFig1	0.346	2	x:1024, y:1024, z:60, channels:32	1.58 µs	727	75 µm	405	0.3% @ 405 nm	3.75
SupFig1	0.371	2	x:1024, y:1024, z:62, channels:32	3.15 µs	820	41 µm	458/561	2% @ 561 nm	3.88
SupFig1	0. 865	2	x:512, y:512, z:70,	1.58 µs	800	75 µm	488/561	3% @ 561 nm	10.97

			channels:32, time:10					2.6% @ 488 nm	
SupFig1	0. 865	2	x:512, y:512, z:70, channels:32, time:10	1.58 µs	800	75 µm	488/561	3% @ 561 nm 2.6% @ 488 nm	21.94
SupFig1	0. 865	2	x:512, y:512, z:70, channels:32, time:10	1.58 µs	800	75 µm	488/561	3% @ 561 nm 2.6% @ 488 nm	43.88

Supplementary Table 2: Processing time comparison SEER vs Independent Component Analysis (scikit-learn implementation) for Figures 4-7.

	Processing Time		
	**SEER [sec]**	**ICA (3c) [sec]**	**Speed Up [folds]**
**Figure 4**	0.44	3.45	7.9
**Figure 5**	6.27	256.86	41.0
**Figure 5 subset**	2.89	77.82	26.9
**Figure 6**	1.49	33.58	22.6
**Figure 7**	0.52	10.19	19.5

Supplementary Table 3: Average colorfulness, contrast and sharpness score across figures 4-7 for different visualization methods

	Average Score		
	Colorfulness	Contrast	Sharpness
**Gauss. Def.**	2.11	53.84	10.83
**Gauss r=.1**	2.06	52.19	10.60
**Gauss r=.2**	1.97	59.57	11.17
**Gauss r=.3**	1.96	60.00	11.20
**Peak Wav.**	2.29	58.61	11.14
**SEER h=1**	2.34	66.32	11.40
**SEER h=2**	2.34	65.15	11.40

Supplementary Table 4: Color Quality Enhancement score for datasets in figures 4-7. Parameters calculations are reported in methods section.

	Color Quality Enhancement			
	Figure 4	Figure 5	Figure 6	Figure 7
Gauss. Def.	39.47	22.72	58.37	46.64
Gauss r=.1	38.26	16.39	61.29	46.34
Gauss r=.2	43.55	30.11	62.53	46.71
Gauss r=.3	43.07	30.26	63.89	46.89
Peak Wav.	43.15	29.09	61.70	47.27
SEER h=1	45.72	31.08	72.87	53.18
SEER h=2	48.38	32.37	69.33	49.58

Supplementary Table 5: Primer list for plasmid constructions

#	Name	Sequence
**1**	attB1-Cerulean-P2A1-F	ggggacaagtttgtacaaaaaagcaggctaccatggtgagcaagggcgaggagctg
**2**	attB1-Cerulean-P2A1-R	ggttctcctccacgtctccagcctgcttcagcaggctgaagttagtagctccgcttcccttgtacagctcgtccatgccg
**3**	P2A1-CreERT2-attB2-F	caggctggagacgtggaggagaaccctggacctaatttactgaccgtacaccaaaatttg
**4**	P2A1-ERT2CreERT2-attB2-R	ggggaccactttgtacaagaaagctgggtaggagtgcggccgctatcaagc
**5**	attB2r-WPRE-attB3-F	ggggacagctttcttgtacaaagtggggtcaacctctggattacaaaatttgtg
**6**	attB2r-WPRE-attB3-R	ggggacaactttgtataataaagttggtgcggggaggcggcccaaagg
**7**	attB1-mKO2-F1	ggggacaagtttgtacaaaaaagcaggcttcaccatggtgagtgtgattaaaccagag
**8**	mKO2-attB2-R1	ggggaccactttgtacaagaaagctgggttttaatgagctactgcatcttctacctgc

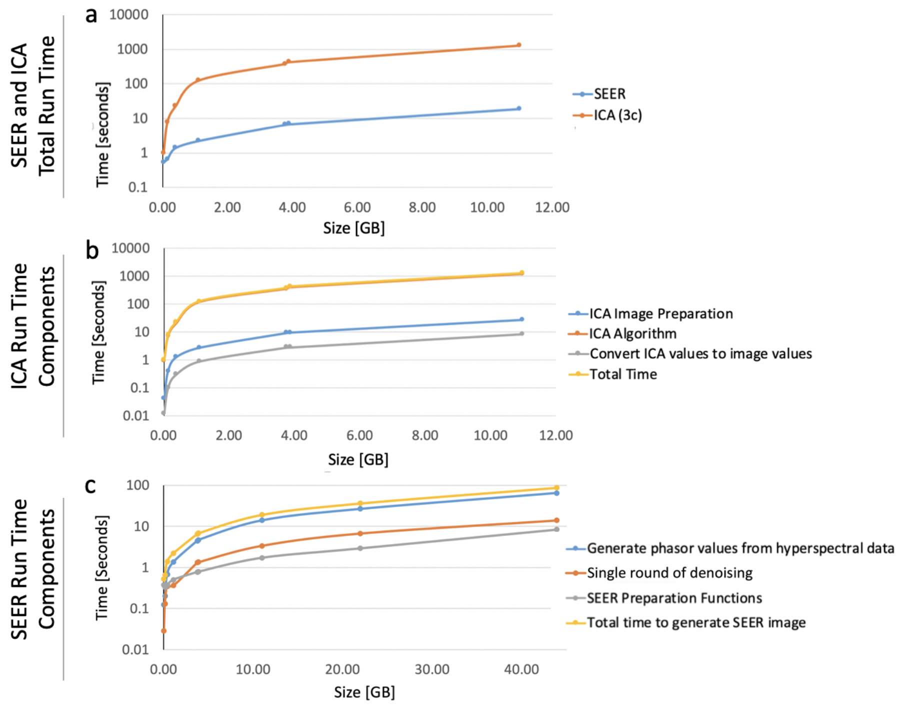

Supplementary Figure 1. Computational time comparison of SEER and ICA for different file sizes. (**a**) HySP and ICA run times (plot in log scale) were measured on a HP workstation with two 12 core CPUs, 128 GB RAM, and 1TB SSD. SEER run times were measured within a modified version of the software. ICA run times were measured using a custom script and the FastICA submodule of the python module, scikit-learn. Timers using the perf_counter function within the python module, time, were placed around specific functions corresponding to the calculations required for the creation of SEER maps in HySP and extracting individual component outputs from the custom ICA script. Data size varies from 0.02-10.97GB, with constant number of bands (32 bands, 410.5 nm to 694.9 nm with 8.9 nm bandwidth) corresponding to a range of $2.86 \cdot 10^5$-$1.83 \cdot 10^8$ spectra. ICA testing was limited to 10.97GB maximum as for higher values the RAM requirements exceeded the 128GB available on our workstation. (**b**) For the custom ICA script, timers were placed to measure the time to reshape the hyperspectral data for ICA input, to run the ICA algorithm, and to convert values of the ICA components into image intensity values, reaching minutes of computation at just 1.1GB (plot in log scale) . (**c**) For HySP, timers were placed to measure the generation of the phasor values from hyperspectral data, including initial calculations of the real and imaginary components (g and s) and creation of the phasor plot histogram. A timer was also placed around all preparatory functions required for on-the-fly creation of SEER maps. The more memory-efficient phasor process allowed us to compute datasets of size 0.02-43.9GB, corresponding to a range of $2.86 \cdot 10^5$-$7.34 \cdot 10^8$ spectra (plot in log scale) .

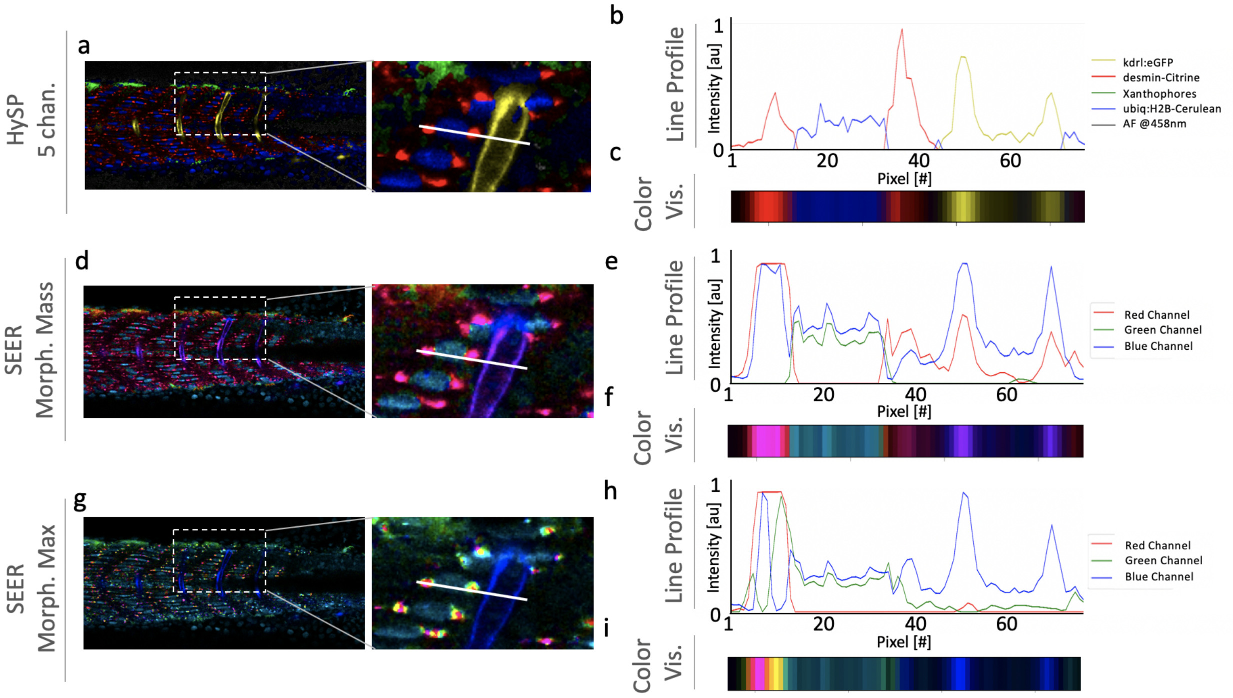

Supplementary Figure 2. Comparison of SEER with visualized HySP[1] results. Here we show a zebrafish embryo *Tg(kdrl:eGFP); Gt(desmin-Citrine);Tg(ubiq:H2B-Cerulean)* labelling respectively vasculature, muscle, and nuclei. Live imaging with a multi-spectral confocal microscope (32-channels) using 458nm excitation. Single plane slices of the tiled volume are rendered with SEER maps (3 channel, RGB) and compared to rendering of the same dataset analyzed with HySP (here 5 channels)[1]. (**a**) Rendering of a 5 channel HySP analyzed dataset, the dashed box is expanded in the zoomed-in portion of panel a with its (**b**) line profile to the right along the solid line all 5 separate channels, eGFP, Citrine, Cerulean, Pigments and autofluorescence at 458nm. (**c**) Visualization of the 5 channel dataset as a blended RGB, similarly to how it appears on a screen. The (**d**) morphed mode center of mass visualization shows patterns in accordance with HySP with a differently color coded (**e**) line profile along the solid line in panel d, which shows intensities in the 3 R,G,B channels of the image. The profiles of the single R,G,B do not match the unmixed HySP profiles in panel b. However, (**f**) color visualization of the same line plot (as R,G,B vectors), shows patterns in accordance to the on-screen visualization of HySP unmixed data. Similarly, (**g**) morphed mode max visualization shows an image in accordance to the rendered HySP analyzed data in panel a with its (**h**) line profile along the solid line of the zoomed-in portion of panel g being comparable to both the HySP 5 separate channels and the R,G,B profiles of the different morphed center of mass map in panel e. (**i**) The color on-display visualization of the RGB intensities in g reveals different color features as those of the HySP unmixed channels (panel b).

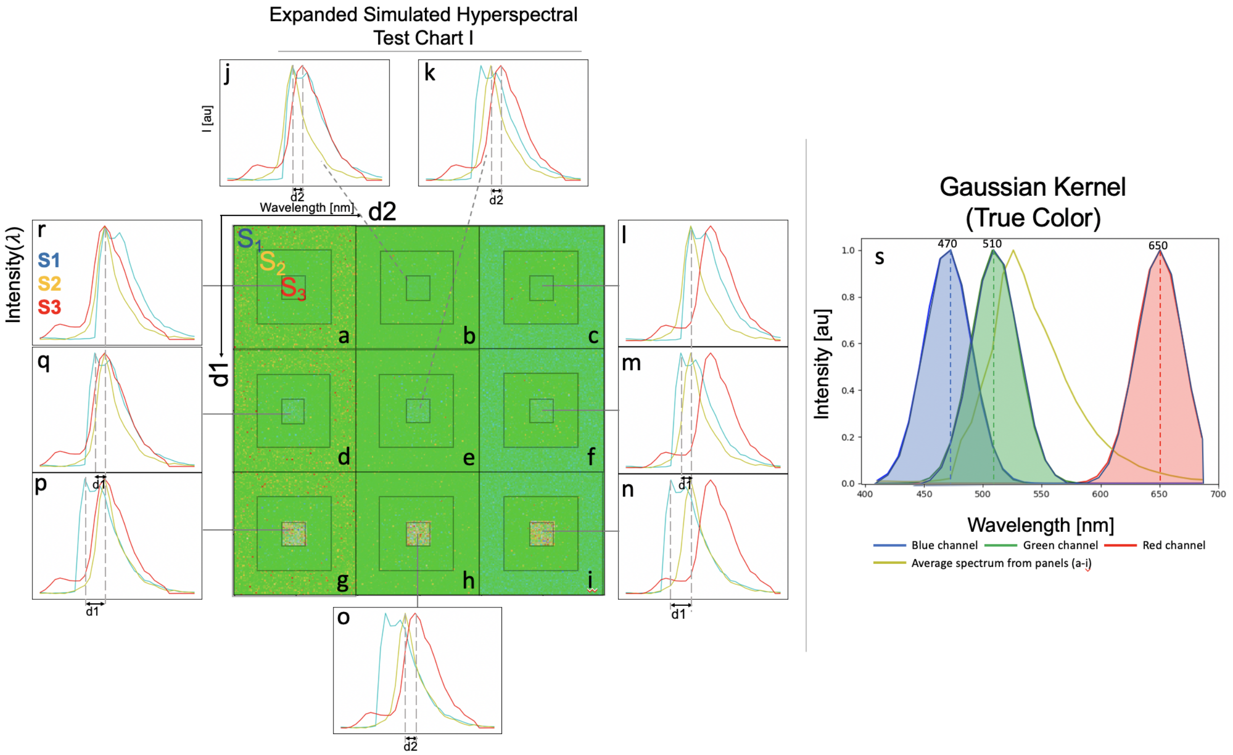

Supplementary Figure 3. Simulated Hyperspectral Test Chart I rendered in TrueColor shows nearly indistinguishable spectra. The simulation is represented here in "TrueColor RGB" (Methods). S_1, S_2, and S_3 spectra acquired respectively from CFP, YFP, RFP zebrafish embryos are used to generate a (**a-i**) 3-by-3 Simulated Hyperspectral Test Chart. In each panel (**a-i**) of the chart, three spectra ($S_1$to S_3) are represented as concentric squares (see panel **a**) outer: S1 - blue, intermediate: S2 - yellow, middle: S3 - red spectra respectively) . The spectrum S_2(intermediate square in each panel) is kept unchanged in all panels. The maximum of spectrum $S_1$is shifted by d1 (-2 wavelength bins, -17.8 nm steps) with respect to the fixed spectrum S_2maximum. S_3max value is shifted by d2 (2 wavelength bins, 17.8nm nm steps) respect to S_2maximum. The changes are applied for 2 steps along the vertical (d1) and horizontal (d2) axis of the center panel assembly (**a-i**), starting from d1=d2=0 (panel **a**). The spectra utilized in each panel (**a-i**) are represented in panels **j-r**. Each plot (**j-r**) represents the averaged normalized S_1-S_3spectra as 32 wavelength bins, 8.9nm bandwidth, 410-695 nm detection. Each panel has different visual contrast but is generally difficult to distinguish by eye due to significant overlap in spectra. (**s**) R,G,B channels used in the Gaussian Kernel for True color representation (red, green, blue lines) and average spectrum for panels (**a-i**) (yellow line) for reference.

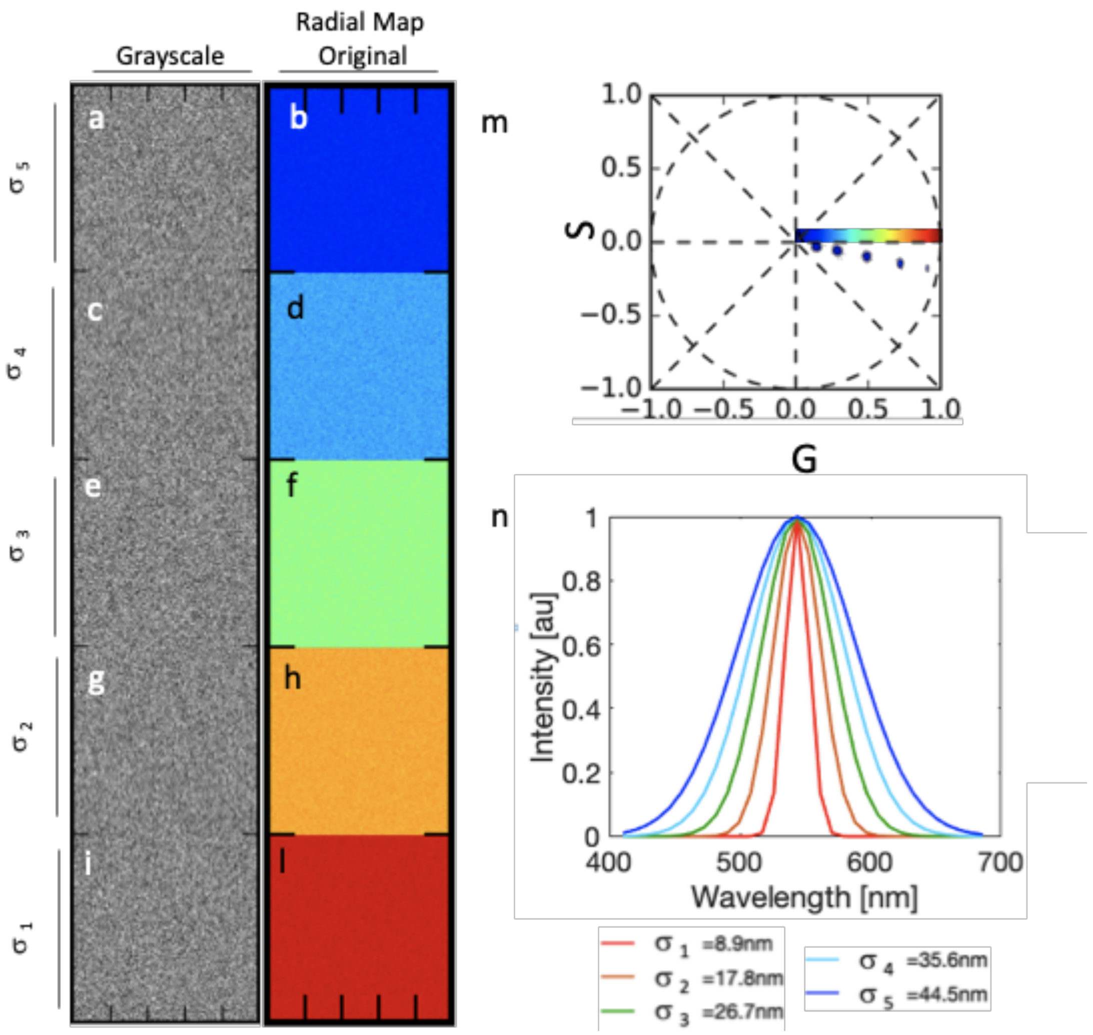

Supplementary Figure 4. Effect of spectral shape with constant intensities on radial map in absence of background. This simulation shows spectra with Gaussian shape and different standard deviations on using 32 wavelength bins, 8.9nm bandwidth, and a 410-695 nm range in the absence of background. All spectra are centered on 543nm (channel 16) and the integral of intensities is kept constant. (**a-l**) For each value of the standard deviation, a grayscale image and SEER visualization are presented. The map used is the Radial map centered on the origin and extended to the border of the phasor plot. A color reference is added in the phasor plot (**m**). Clusters on the phasor plot are distributed along the radius, with distance from the origin inversely proportional to the standard deviation.

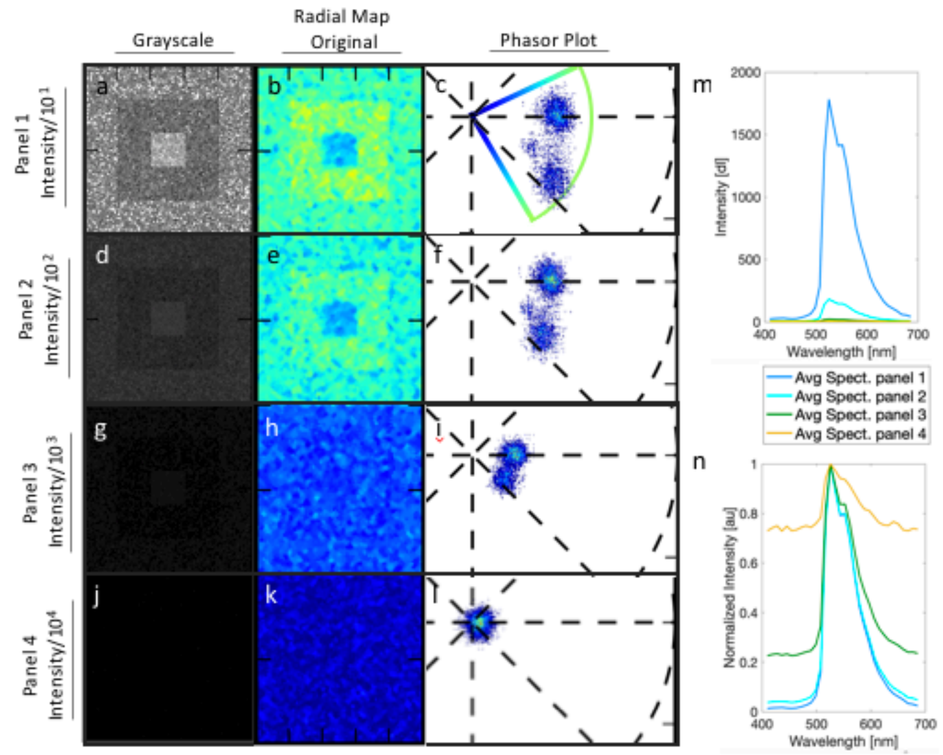

Supplementary Figure 5. Effect of spectrum intensity in presence of background on radial map. In this simulation, the first panel (top-left) of the Simulated Hyperspectral Test Chart (Supplementary Figure 3) is reduced in intensity by a factor of 10^1- 10^4 (panel 1-4 respectively) in the presence of a constant background. Background with an average intensity of 5 digital levels was generated in Matlab; poissonian noise was added using the poissrnd() function. Grayscale images (**a,d,g,j**) are scaled by (**a**) factor of 10, (**d**) factor of 10^2, (**g**) factor of 10^3, (**j**) factor of 10^4. Radial map (original) visualization shows a shift of panel colors toward blue with the decreasing intensities (**b,e,h,k**). The phasor plots (**c,f,i,l**) (harmonic n=2) show a radial shift of the clusters toward the origin. Radial map reference is added in (**c**). (**m**) Absolute intensities plot shows the average spectrum for the four panels, maximum peak values are 1780, 182, 23, 7 digital levels (panel 1-4 respectively). The normalized intensity spectra (**n**) show an apparent broadening of the shape of spectra with the decreasing signal-to-noise.

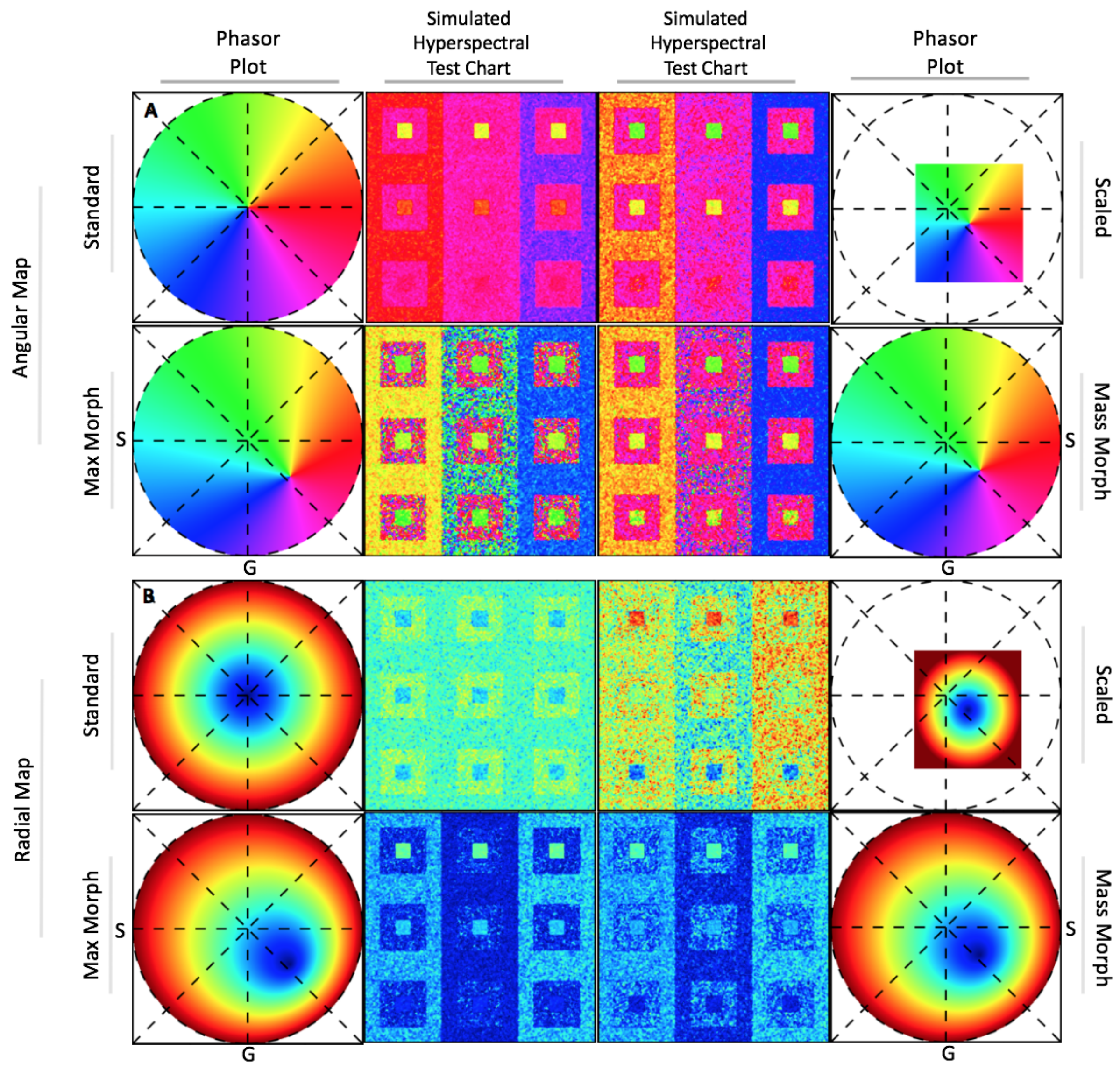

Supplementary Figure 6. Radial and Angular reference map designs and modes differentiate nearly indistinguishable spectra (Simulated Hyperspectral Test Chart I) (Supplementary Figure 3). We present 4 different modes that can be applied for each map. Here second harmonic is utilized for the calculations. Angular map (**a**) and Radial map (**b**) in Standard mode, Scaled mode, Max Morph mode and Mass Morph mode. In Standard mode, the reference map is centered at the origin and limited by the phasor unit circle. In Scaled mode, the reference map adapts to the phasor plot histogram, changing its coordinates to wrap around the edges of the phasor clusters and enhancing contrast of the chosen map properties. In Max Morph mode, the map is centered on the spectrum with highest frequency of appearance in the phasor histogram. This mode improves sensitivity by using statistical frequency bias. In Mass Morph mode, the map is centered on the weighted center of the phasor, enhancing sensitivity for multiple small spectra. Visualizations are presented after 1x spectral denoising.

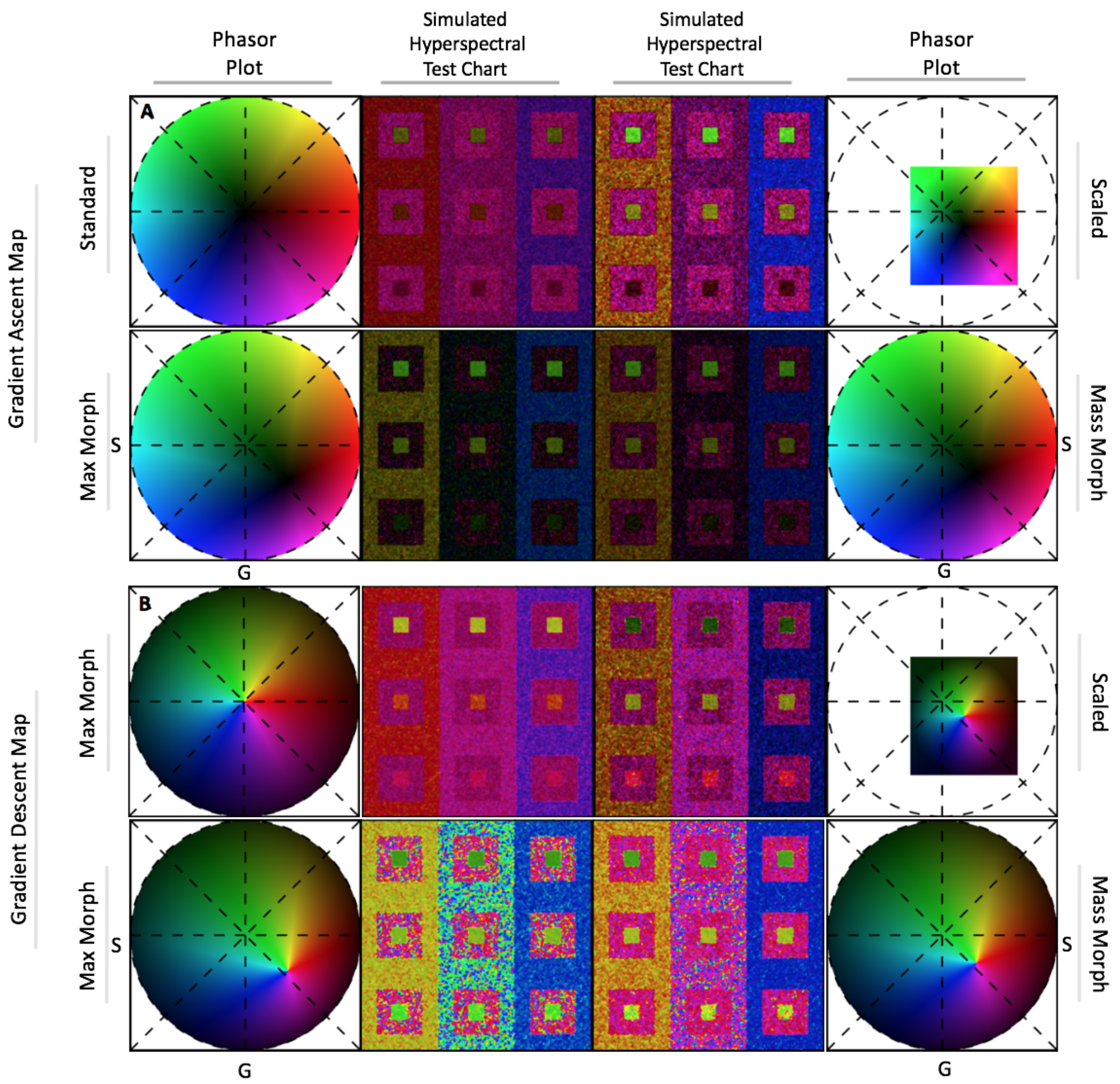

Supplementary Figure 7. Gradient Ascent and Descent reference map designs and modes differentiate nearly indistinguishable spectra (Supplementary Figure 3). Here second harmonic is utilized for SEER. Gradient Ascent map (**a**) and Gradient Descent map (**b**) in Standard mode, Scaled mode, Max Morph mode and Mass Morph mode. The two maps place a focus on very different (Ascent) and similar (Descent) spectra by fading the reference map to dark at the center and edges of the phasor plot unit circle respectively. Visualizations are presented after 1x spectral denoising.

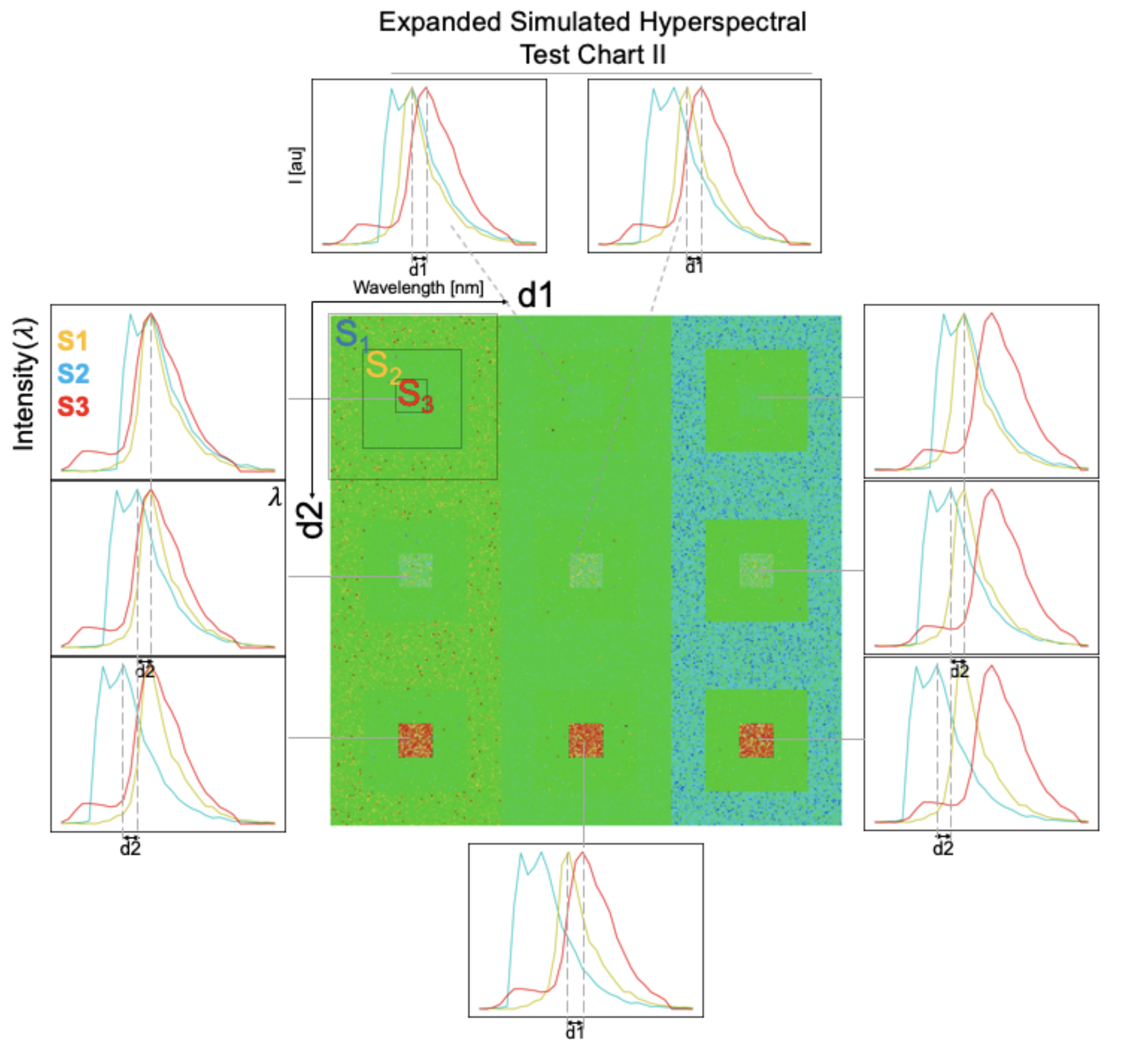

Supplementary Figure 8. Simulated Hyperspectral Test Chart II and its standard overlapping spectra. Simulated SHTC II was generated from the same zebrafish embryo datasets and same design used in SHTC1 (Supplementary Figure 3) utilizing CFP, YFP and RFP labeled samples and 3-by-3 block chart, with each block subdivided into 3 regions corresponding to spectra S_1, S_2, and S_3 . The aim is to test scenarios with less overlapping spectra. We change the shifting distance in this simulation to be d1 (-3 wavelength bins, -26.7nm nm steps) and d2 (3 wavelength bins, 26.7nm steps). The channels used in the Gaussian Kernel for TrueColor RGB representation here were 650nm, 510nm, 470nm which respectively represent R, G, B. The concentric squares in the lower right side of the simulation are separated by a peak-to-peak distance of 53.6nm , with outer and inner concentric squares well separated by 106.8nm. This distance is similar to the emission gap between CFP (475nm EM) and tdTomato (581nm). Under these spectral conditions most methods are expected to perform well.

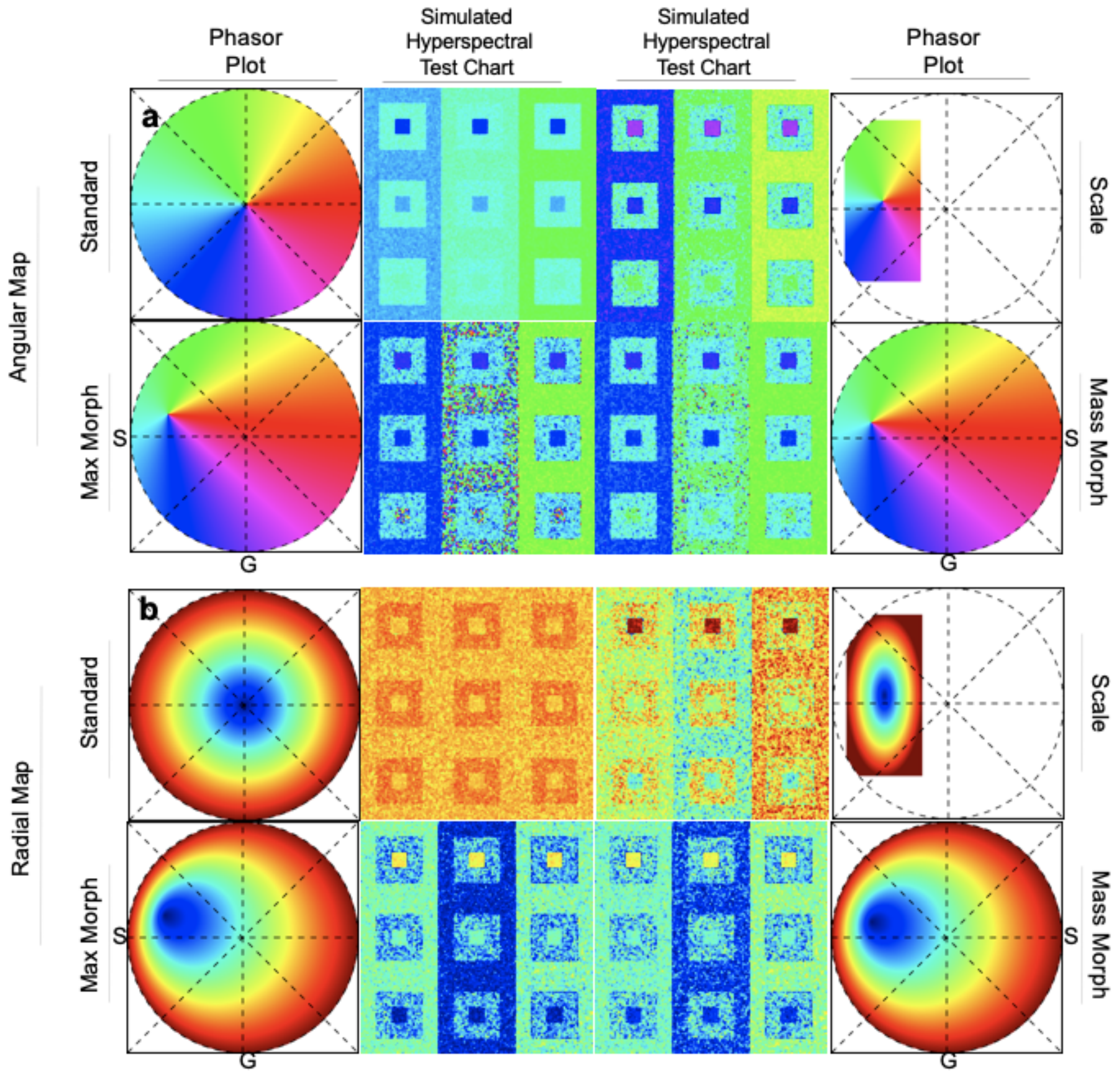

Supplementary Figure 9. Radial and Angular reference map designs and modes rendering standard overlapping spectra (Simulated Hyperspectral Test Chart II) (Supplementary Figure 2). Here first harmonic is utilized for SEER Angular map (**a**) and Radial map (**b**) in Standard mode, Scaled mode, Max Morph mode and Mass Morph mode are here applied to the standard overlapping spectra simulation. The reference maps show improved contrast consistently among different modalities. Visualizations are presented after 1x spectral denoising.

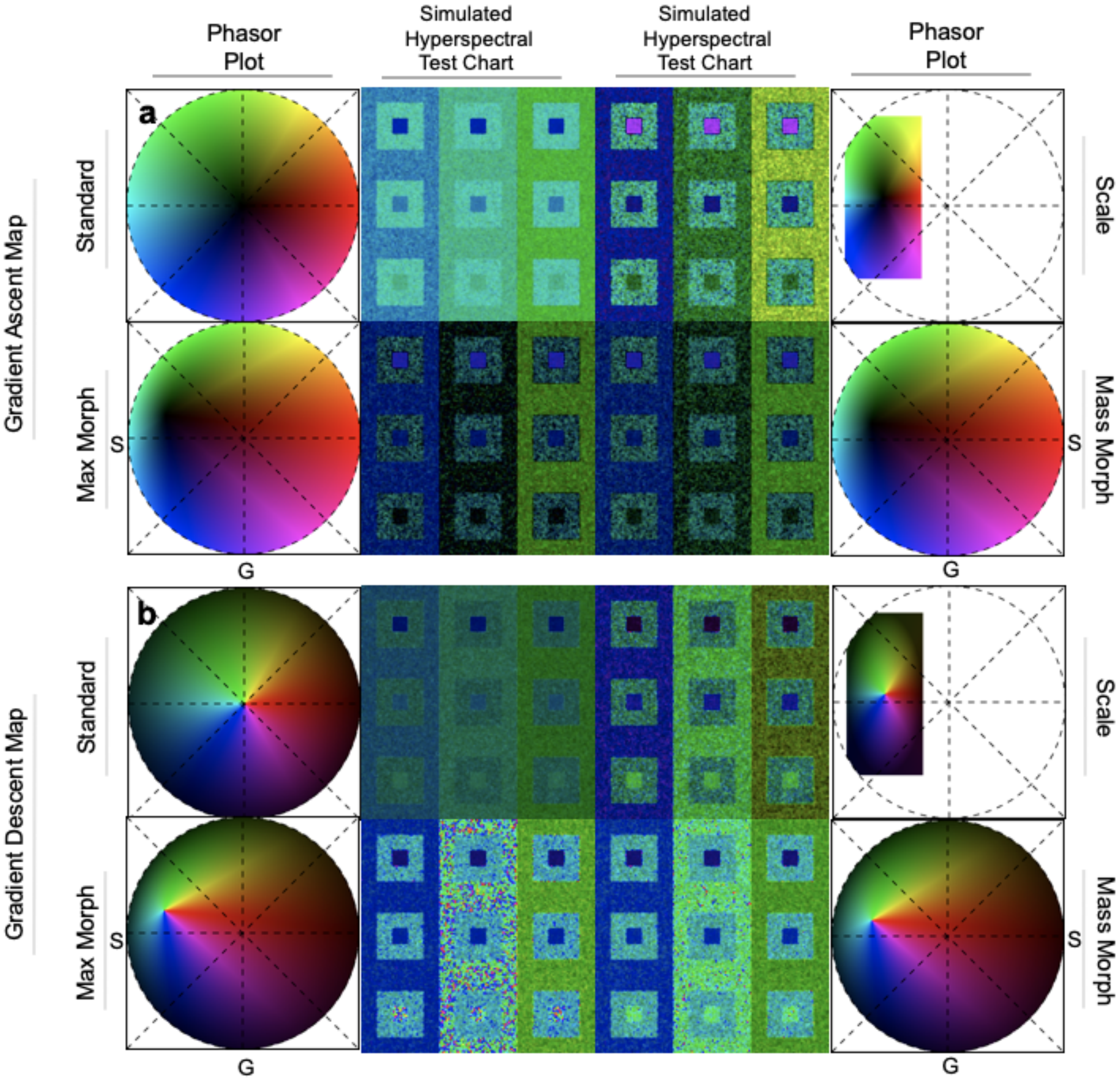

Supplementary Figure 10. Gradient ascent and descent reference map designs and modes differentiation of standard overlapping spectra (Simulated Hyperspectral Test Chart II). Here first harmonic is utilized for SEER Gradient Ascent map (**a**) and Gradient Descent map (**b**) in Standard mode, Scaled mode, Max Morph mode and Mass Morph mode. The reference maps provide enhanced visualization even in the scenario of spectra overlapping at similar level to commonly used fluorescent proteins. Visualizations are presented after 1x spectral denoising.

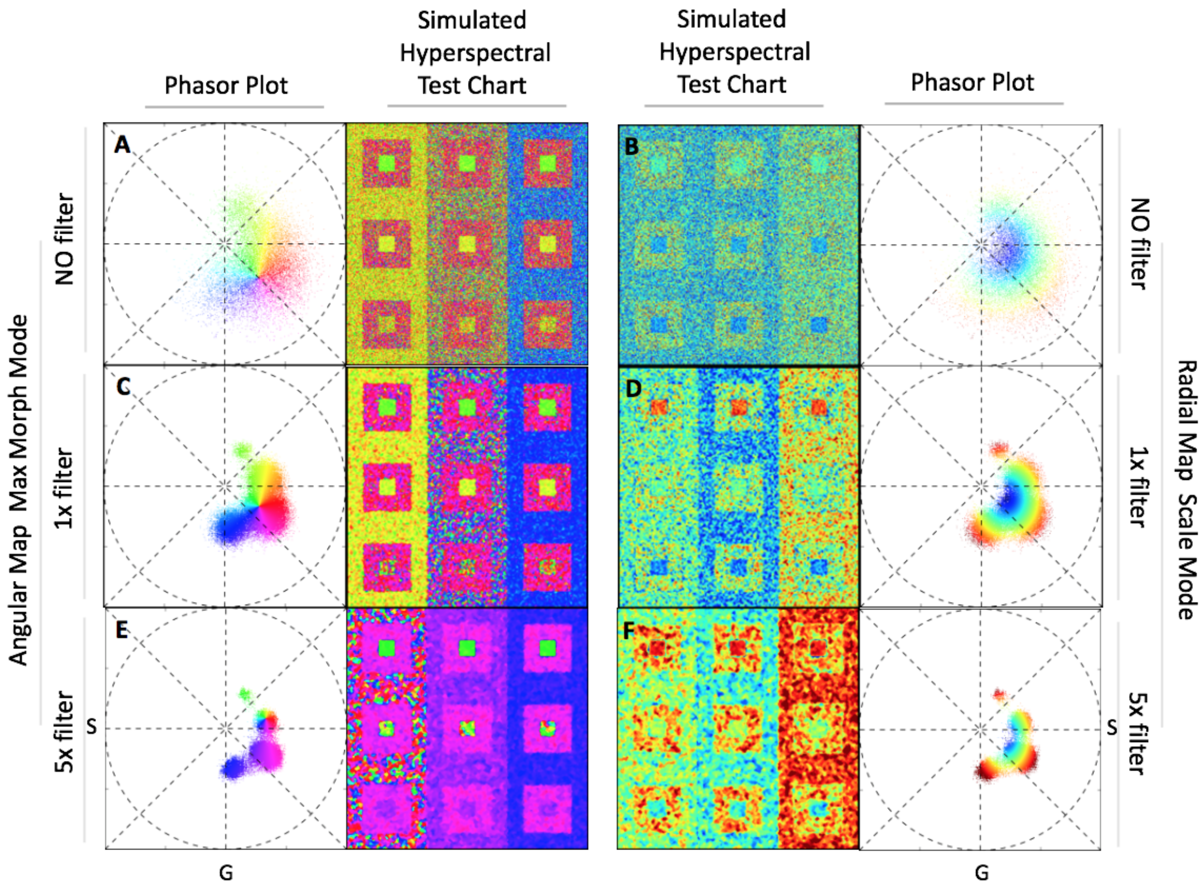

Supplementary Figure 11. Spectral denoising effect on Angular and Radial maps visualization of standard overlapping spectra (Simulated Hyperspectral Test Chart II, Supplementary Figure 8). Phasor spectral denoising affects the quality of data along the spectral dimension, without changing intensities. Here second harmonic is utilized for calculations. Noisy data appears as a spread cluster on the phasor, here shown overlaid with the (**a**) Angular map and (**b**) Radial map, with the overlaid visualization exhibiting salt and pepper noise. (**c**, **d**) When denoising is applied on the phasor, the cluster spread is reduced, providing greater smoothing and less noise in the simulated chart. (**e**, **f**) Increasing the number of denoising filters results in a clearer distinction between the three spectrally different areas in each block of the simulation. (**a**, **c**, **e**) In Max Morph Mode, each denoising filter introduces a shift of the apex of the map, changing the reference center of the color palette (**b**, **d**, **f**) In Scale Mode, the less scattered phasor cluster makes maximum use of the reference maps, enhancing the contrast (**d**, **f**) of the rendered SHTC.

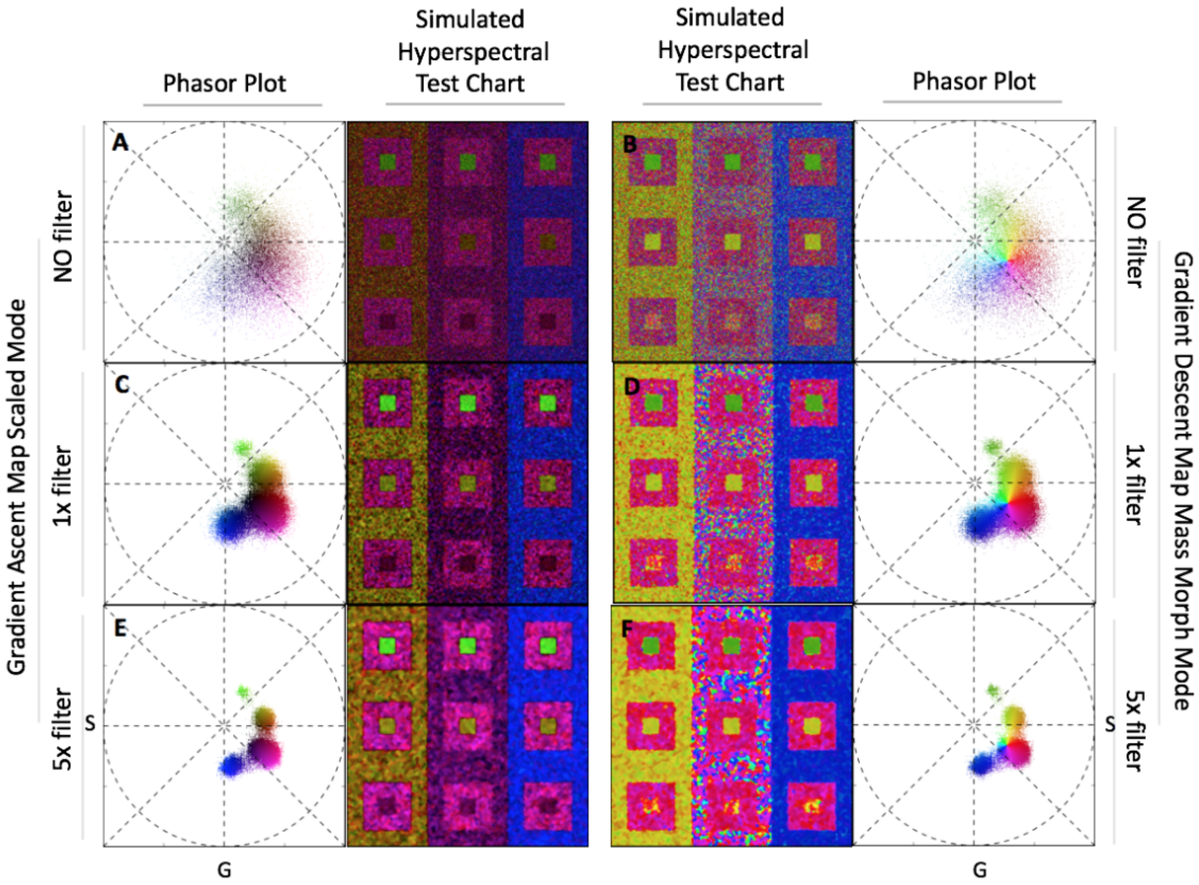

Supplementary Figure 12. Spectral denoising effect on Gradient Ascent and Descent maps visualization of standard overlapping spectra (Simulated Hyperspectral Test Chart II, Supplementary Figure 8). The phasor spectral denoising principle described in (Supplementary Figure 11) applies to different reference maps. In this case (**a**) Gradient Ascent map in Scaled mode and (**b**) Gradient Descent in Mass Morph mode are overlaid to the scattered phasor representation of a standard overlapping spectrum SHTC. The denoising filter removes outliers along the spectral dimension while preserving intensities. (c, d) The phasor cluster spread is reduced after filtering, resulting in spectral smoothing of the images affected by noise. Due to the changes in phasor cluster spread after filtering, the map reference for the Gradient Ascent map has an increased brightness in comparison to its non-filtered representation (chart panel in **a** and **b**). (**e**, **f**) The rendered SHTC after multiple denoising passes has higher intensity, which simplifies distinction of subtle differences in spectra. (b, d, f) The denoising filter does not change the clusters' center of mass, therefore the apex of the reference map remains unchanged after filtering. However, the filters play a role in reducing Poisson noise in the dataset, converging to a stable value after 5x filtering. The representation shows more uniformity in the concentric squared areas of within each block, which are simulated using the same spectrum. The edges of these squares are now more sharp and easier to detect, suggesting the combination of SEER and phasor denoising can play an important role in simplifying image segmentation.

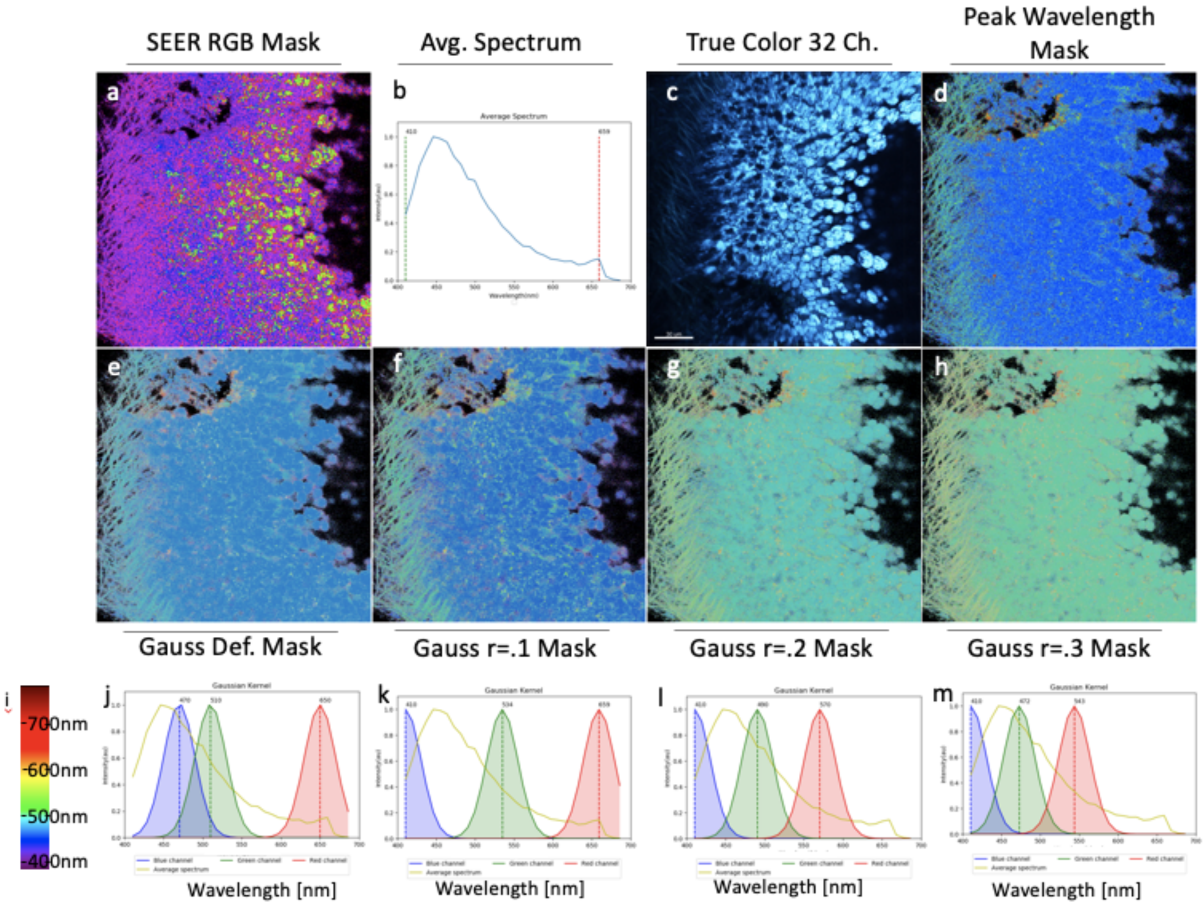

Supplementary Figure 13. Visualization comparison for autofluorescence with other RGB standard visualizations. The visualization of unlabeled freshly isolated mouse tracheal explant (Figure 4) is shown here with different standard approaches. Details for these visualizations are reported in the Methods section. (**a**) SEER RGB mask obtained using gradient descent morphed map; this mask shows the colors associated by SEER to each pixel, without considering intensity. (**b**) Average spectrum for the entire dataset. (**c**) TrueColor 32 channels maximum intensity projection (**d**) Peak wavelength RGB mask. (**e**) Gaussian Default Kernel with RGB centered respectively at 650nm, 510nm and 470nm. (**f**) Gaussian Kernel at 10% threshold, RGB values centered at 659nm, 534nm and 410nm. (**g**) Gaussian Kernel at 20% threshold, RGB values centered at 570nm, 490nm and 410nm. (**h**) Gaussian kernel at 30% threshold, RGB values centered at 543nm, 472nm and 410nm. (**i**) wavelength-to-RGB color representation for Peak Wavelength mask in panel d. A representation of the RGB visualization parameters is reported in the following sub plots (**j**) kernel used for panel e, average spectrum of the dataset (yellow plot), (**k**) kernel used for panel f, average spectrum of the dataset (yellow plot), (**l**) kernel used for panel g, average spectrum of the dataset (yellow plot), (**m**) kernel used for panel h, average spectrum of the dataset (yellow plot).

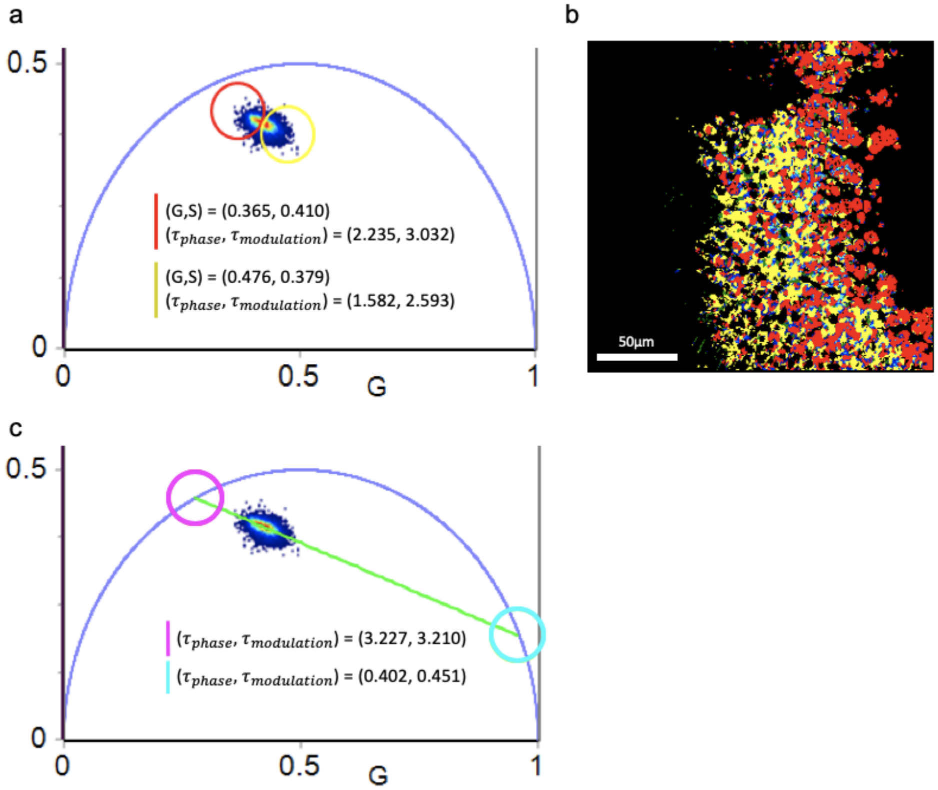

Supplementary Figure 14. Phasor Fluorescence Lifetime Imaging Microscopy (FLIM) of unlabeled freshly isolated mouse tracheal explant. (**a**) Phasor FLIM representation of fluorescence lifetime data for unlabeled freshly isolated mouse tracheal explant acquired in frequency domain utilizing a 2-photon fluorescence microscope (LSM 780, Zeiss, Jena) tuned at 740nm, coupled with an acquisition unit with Hybrid Detectors (FLIM Box, ISS, Urbana-Champaign). The selected regions correspond to more Oxidative Phosphorylation phenotype (red circle) more Glycolytic phenotype (yellow circle). (**b**) FLIM segmented image corresponding to the selection performed on phasor (a) where cells in apical layer exhibit Oxidative Phosphorylation phenotype compared to cells in basal layer with a Glycolytic phenotype.(**c**) The line joining free and bound NADH in the phasor plot is known as the “metabolic trajectory”, and a shift in the free NADH direction is representative of a more reducing condition and a glycolytic metabolism, while a shift towards more bound NADH is indicative of more oxidizing conditions and more oxidative phosphorylation, as described in previous studies[2–5].
The extremes of the metabolic trajectory are the lifetimes for NADH free and bound. The parameters for lifetime (τ phase and modulation) are in line with those reported in literature (0.4ns free and 1.0-3.4 ns bound)[3–8]

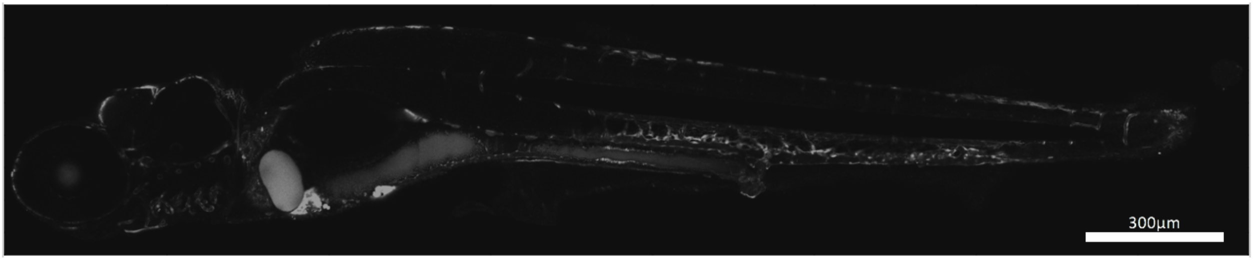

Supplementary Figure 15. Gray scale visualization of a single fluorescence label against multiple autofluorescences. Monochrome representation of the average spectral intensity for a single optical section of Tg(*fli1:mKO2)* (pan-endothelial fluorescent protein label) zebrafish presenting intrinsic signal arising from the yolk and xanthophores (pigment cells). Dataset was acquired using a confocal microscope in multi-spectral mode (LSM 780, Zeiss, Jena) with 488nm excitation. Average intensity was calculated along the spectral dimension and then represented in grayscale.

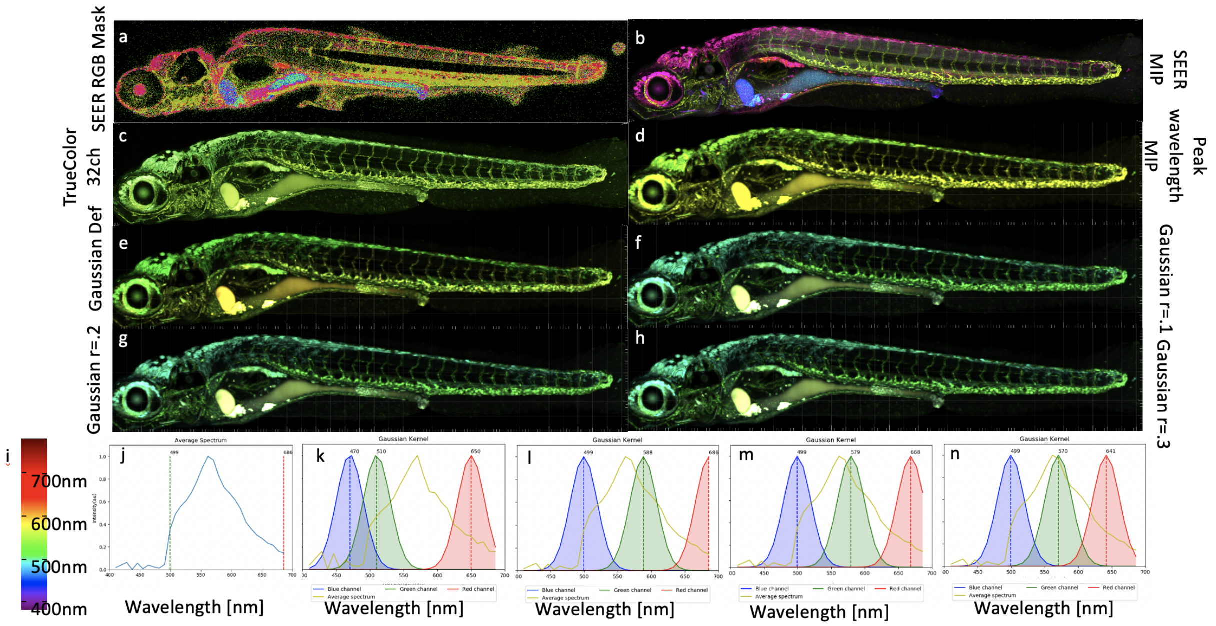

Supplementary Figure 16. Visualization comparison for single fluorescent label with other RGB standard visualizations in presence of autofluorescence. Visualization of *Tg(fli1:mKO2)* (pan-endothelial fluorescent protein label) zebrafish with intrinsic signal arising from the yolk and xanthophores (pigment cells) (Figure 5) is here shown with different standard approaches. Details for these visualizations are reported in the Methods section. (**a**) SEER RGB mask for a single z-plane, obtained using gradient angular map in scaled mode, this mask shows the colors associated by SEER to each pixel, without considering intensity. (**b**) SEER maximum intensity projection (MIP) for the entire volume (**c**) TrueColor 32 channels volume MIP (**d**) Peak wavelength volume MIP. (**e**) Gaussian Default Kernel with RGB centered respectively at 650nm, 510nm and 470nm. (**f**) Gaussian Kernel at 10% threshold, RGB values centered at 686nm, 588nm and 499nm. (**g**) Gaussian Kernel at 20% threshold, RGB values centered at 668nm, 579nm and 499nm. (**h**) Gaussian kernel at 30% threshold, RGB values centered at 641nm, 570nm and 499nm. (**i**) wavelength-to-RGB color representation for Peak Wavelength mask in panel d. A representation of the RGB visualization parameters is reported in (**j**) Average spectrum (blue plot) for the entire dataset with boundaries used for TrueColor 32ch MIP in panel c. (**k**) Kernel used for panel e, average spectrum of the dataset (yellow plot), (**l**) kernel used for panel f, average spectrum of the dataset (yellow plot), (**m**) kernel used for panel g, average spectrum of the dataset (yellow plot), (**n**) kernel used for panel h, average spectrum of the dataset (yellow plot).

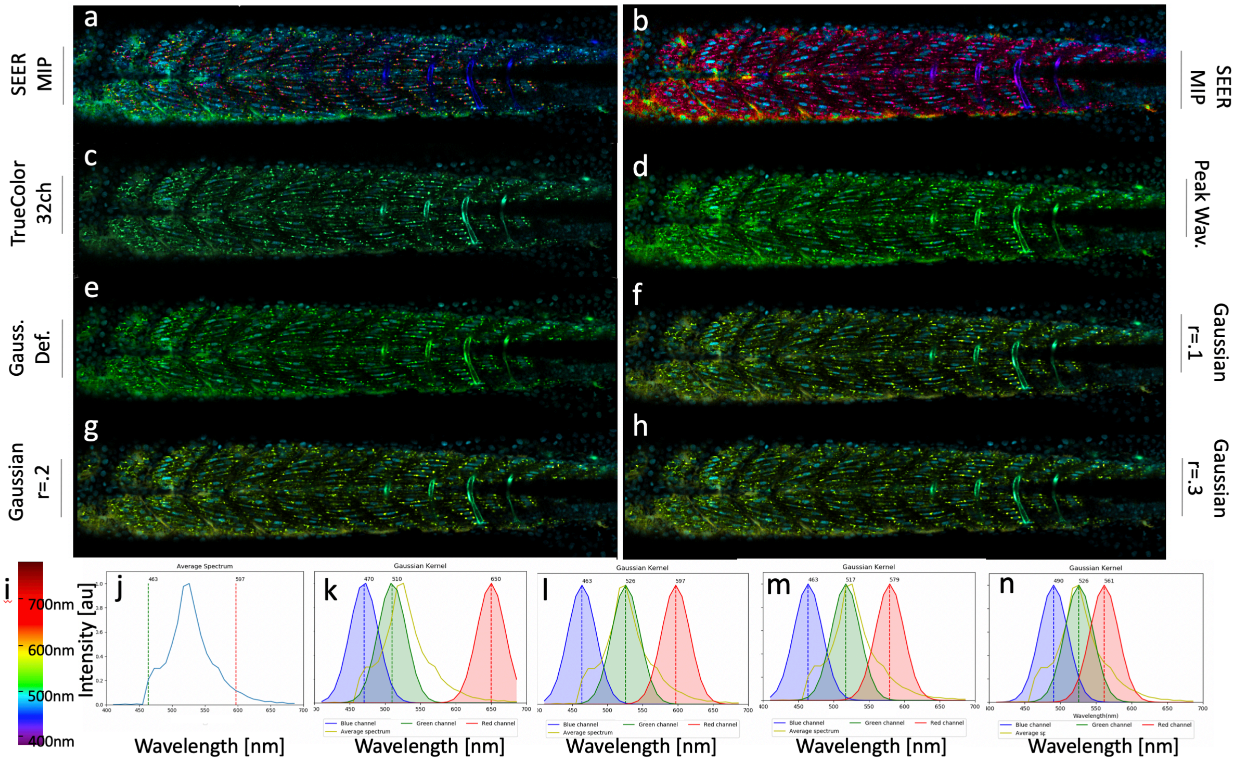

Supplementary Figure 17. Visualization comparison for triple label fluorescence with other RGB standard approaches. Visualization of *Tg(kdrl:eGFP); Gt(desmin-Citrine); Tg(ubiq:H2B-Cerulean)* labelling respectively vasculature, muscle, and nuclei (Figure 6) is shown here with different standard approaches. Details for these visualization are reported in the Methods section. The same slice (here z=3) is shown as a maximum intensity projection (MIP) using: (**a**) SEER gradient descent map in max morph mode, (**b**) SEER MIP angular map mass morph mode, (**c**) TrueColor 32 channels, (**d**) Peak wavelength, (**e**) Gaussian Default Kernel with RGB centered respectively at 650nm, 510nm and 470nm. (**f**) Gaussian Kernel at 10% threshold, RGB values centered at 597nm, 526nm and 463nm (**g**) Gaussian Kernel at 20% threshold, RGB values centered at 579nm, 517 and 463nm (**h**) Gaussian kernel at 30% threshold, RGB values centered at 561nm, 526nm and 490nm. A representation of the RGB visualization parameters is reported in (**i**) wavelength-to-RGB color representation for Peak Wavelength mask in panel d, (**j**) Average spectrum (blue plot) for the entire dataset with boundaries used for TrueColor 32ch MIP in panel c. (**k**) kernel used for panel e, average spectrum of the dataset (yellow plot), (**l**) kernel used for panel f, average spectrum of the dataset (yellow plot), (**m**) kernel used for panel g, average spectrum of the dataset (yellow plot), (**n**) kernel used for panel h, average spectrum of the dataset (yellow plot).

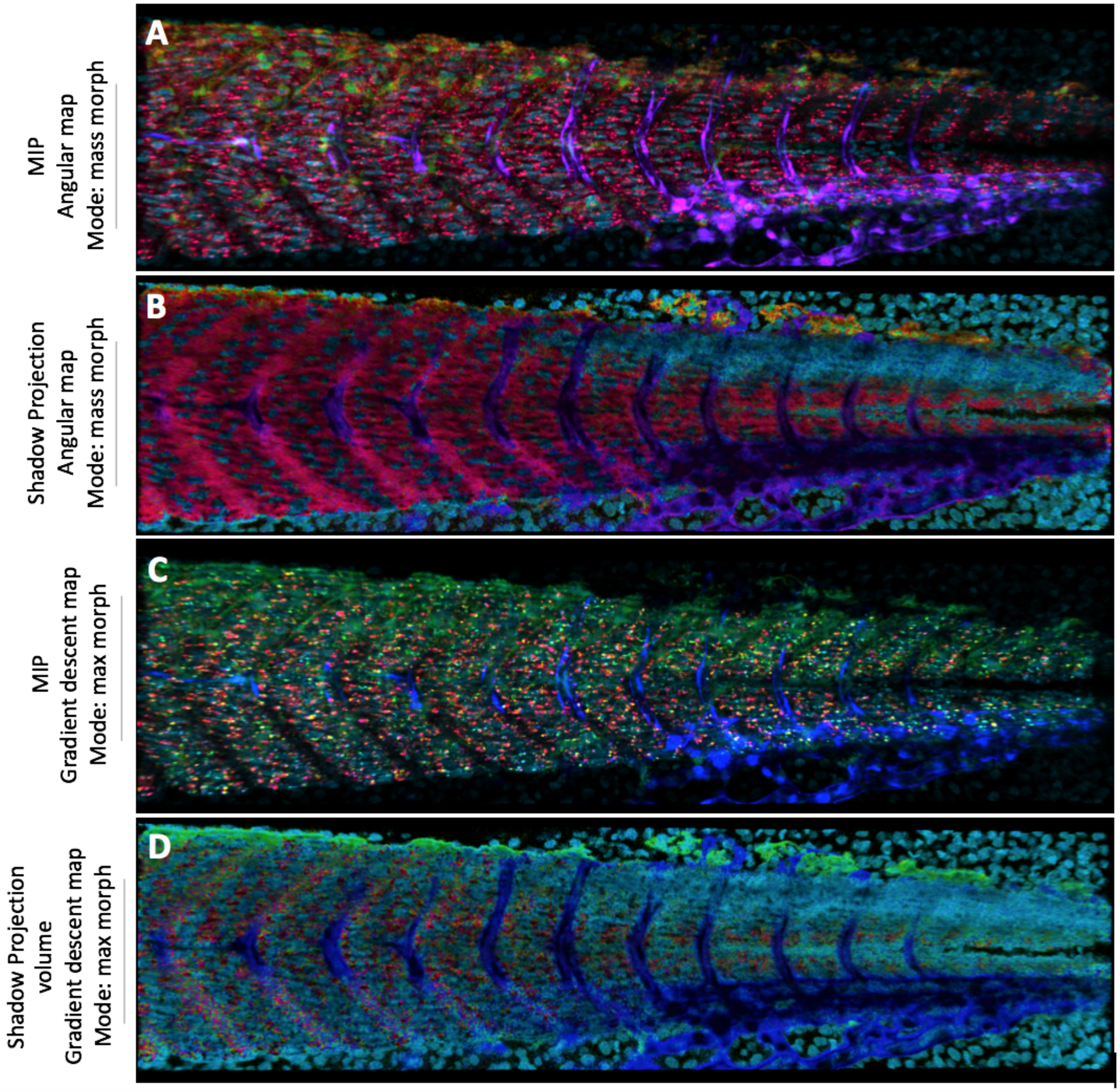

Supplementary Figure 18. SEER of zebrafish volumes in Maximum Intensity Projection (MIP) and Shadow Projection. The capability of SEER to improve visualization of spectral datasets is translatable to 3D visualizations with different visualization modalities. Here we show a zebrafish embryo *Tg(kdrl:eGFP); Gt(desmin-Citrine);Tg(ubiq:H2B-Cerulean)* labelling respectively vasculature, muscle, and nuclei. (**a**) MIP of an Angular map volume with Mass Morph mode. (**b**) The same combination of map and mode is shown using shadow projection. While the volume rendering approaches are different, the spatial distinction between fluorescent labels is maintained. The Gradient Descent map in Max Morph mode is here applied on the same dataset using (**c**) MIP and (**d**) shadow projection. With the Gradient Descent map (**c**) MIP improves contrast for determining spatial distinction between fluorophores. (d) Shadow projection further enhances the location of skin pigments (green).

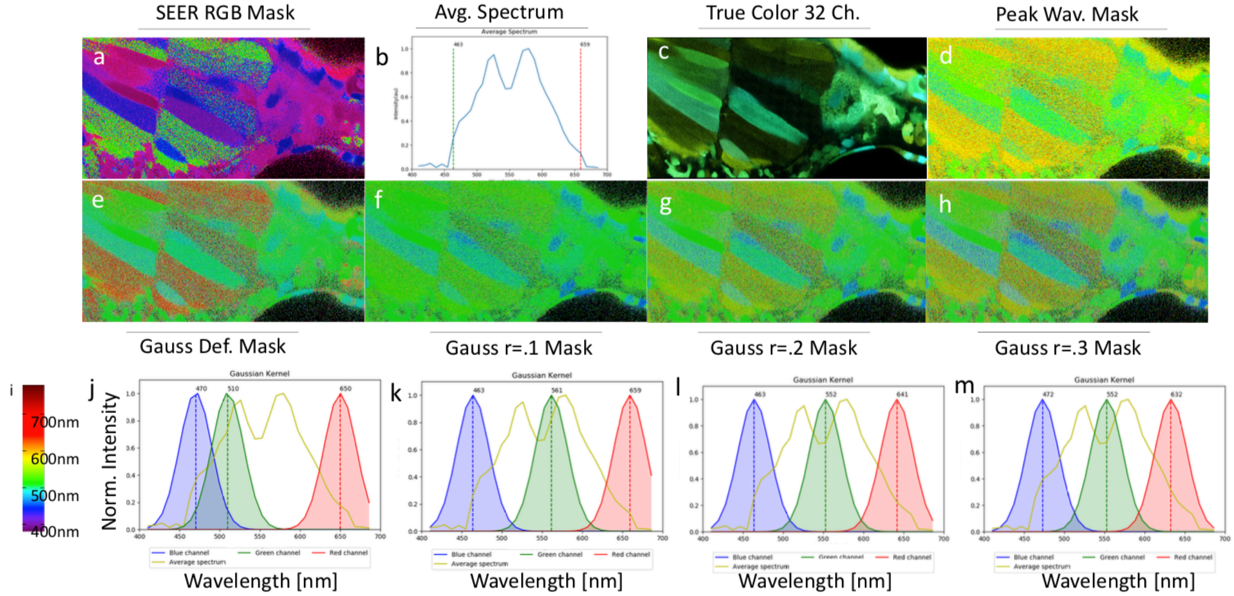

Supplementary Figure 19. Visualization comparison for combinatorial expression with other RGB standard approaches. Visualization of *ubi:Zebrabow* muscle (Figure 7) with different standard approaches. Details for these visualization are reported in the Methods section. The same slice is shown as an RGB mask which represents the color associated to each pixel, independent from the intensity, or as a maximum intensity projection (MIP) using: (**a**) SEER gradient descent map mask in scaled mode, (**b**) Average spectrum (blue plot) for the entire dataset with boundaries used for TrueColor 32ch MIP in panel c. (**c**) TrueColor 32 channels, (**d**) Peak wavelength mask, (**e**) Gaussian Default Kernel with RGB centered respectively at 650nm, 510nm and 470nm. (**f**) Gaussian Kernel at 10% threshold, RGB values centered at 659nm, 561nm and 463nm. (**g**) Gaussian Kernel at 20% threshold, RGB values centered at 641nm, 552nm and 463nm. (**h**) Gaussian kernel at 30% threshold, RGB values centered at 632nm, 552nm and 472nm. (**i**) wavelength-to-RGB color representation for Peak Wavelength mask in panel d. A representation of the RGB visualization parameters is reported in (**j**) kernel used for panel e, average spectrum of the dataset (yellow plot), (**k**) kernel used for panel f, average spectrum of the dataset (yellow plot), (**l**) kernel used for panel g, average spectrum of the dataset (yellow plot), (**m**) kernel used for panel h, average spectrum of the dataset (yellow plot).

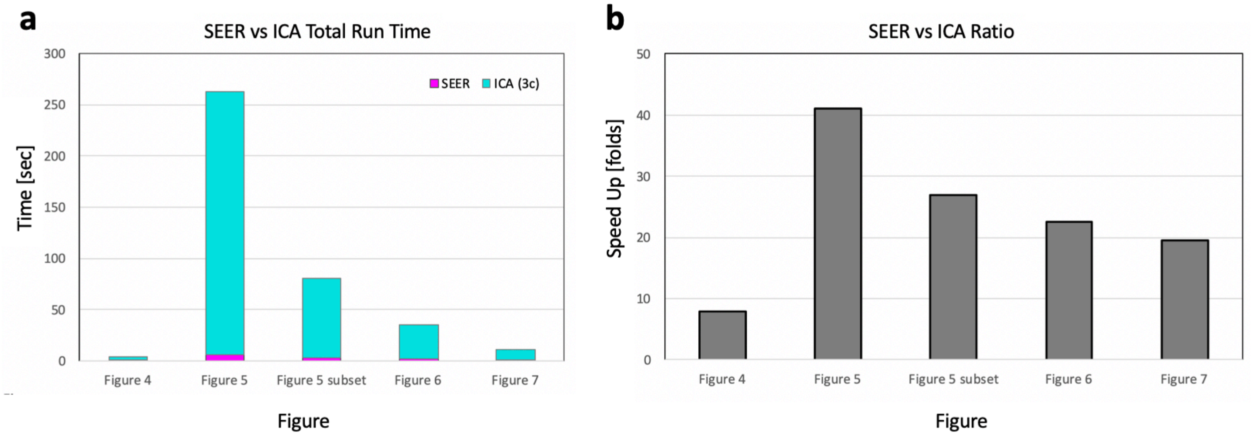

Supplementary Figure 20. Processing speed comparison SEER vs Independent Component Analysis for the datasets of Figures 4-7. Here we compare the processing time between SEER and the FastICA submodule of the python module, scikit-learn. With the same measurement strategy used in Supplementary Figure 1, timers using the perf_counter function within the python module, time, were placed around specific functions corresponding to the calculations required for the creation of SEER maps in HySP and with FastICA. (a) Run time for SEER (magenta) was considerably lower than ICA (3 components) (cyan) in all Figures and their subsets. (b) The speed up was higher for larger z-stack spectral datasets (Figure 5, 41-fold improvement) and reduced for smaller, single spectral images (Figure 4, 7.9-fold improvement). Numerical values for these plots are reported in Supplementary Table 2.

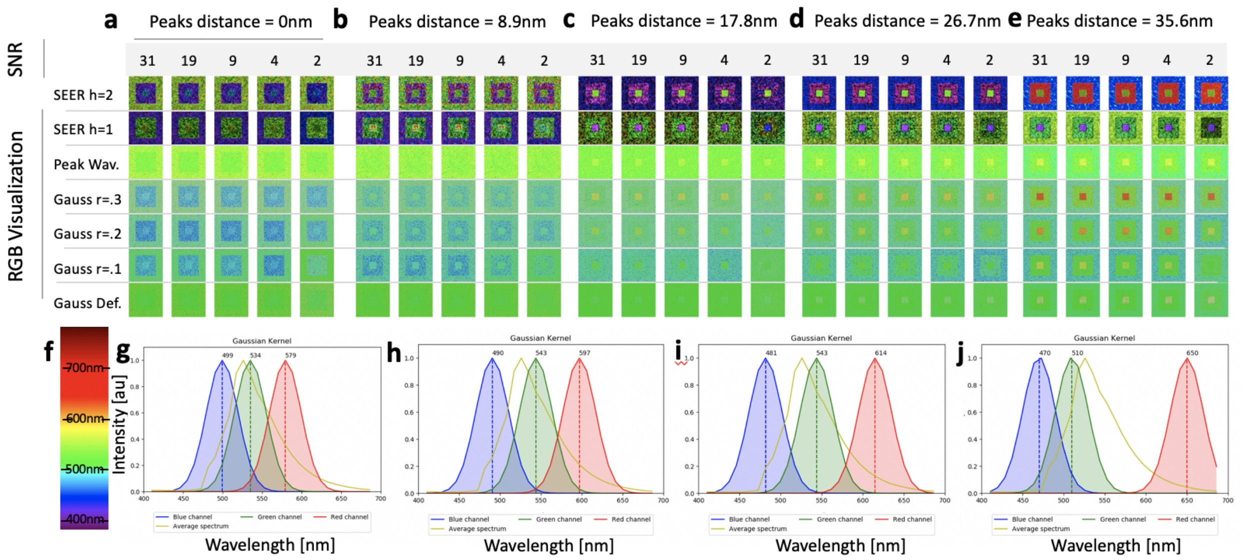

Supplementary Figure 21. RGB Visualization with multiple modalities under different spectral overlap and SNR conditions. In this simulation, the first panel (top-left) of the Simulated Hyperspectral Test Chart (SHTC, Supplementary Figure 3) is reduced in intensity by a factor of $5*10^0$ - $5*10^4$ (panel 1-5 respectively) in the presence of a constant background. Background with average intensity 5 was generated in Matlab, poissonian noise was added using the poissrnd() function obtaining 5 different levels of SNR. (**a,b,c,d,e**) Peak-to-peak distance for the spectra in the middle and outer concentric squares in the SHTC is shifted by units of 8.9nm with respect to the peak of the average spectrum in the intermediate square, which is kept constant in this simulation (similarly to Supplementary Figure 3) starting from distance 0 (a) to 35.6nm (e). For each level of spectral overlap (**a-e**), seven different RGB visualization modalities are presented here for comparison at five different level of SNR. In order from the top row, SEER at harmonic 2 (SEER h=2) and harmonic 1 (SEER h=1), peak wavelength selected (Peak Wav.), Gaussian kernel set at 30% of the spectrum (Gauss r=.3), set at 20% (Gauss r=.2) and at 10% (Gauss r=.1), finally Gaussian kernel set at 650nm, 510nm, 470nm for RGB respectively (Gauss. Def.). (**f**) the wavelength-to-RGB conversion map used for the peak wavelength visualization. (**g**) center wavelength for the R=579nm, G=534nm, B=499nm channels of Gauss r=.3. Average spectrum (yellow) (**h**) center wavelength for the R=597nm, G=543nm, B=490nm channels of Gauss r=.2. Average spectrum (yellow). (**i**) center wavelength for the R=614nm, G=543nm, B=481nm channels of Gauss r=.1. Average spectrum (yellow). (**j**) center wavelength for the R=650, G=510nm, B=470nm channels of Gauss. Def. Average spectrum (yellow). The maps utilized here for SEER were gradient descent in scale mode (a, b, c, d), and center of mass mode (e). Visualization with SEER shows a reasonably constant contrast and color for the different spectra in the simulation at different SNR.

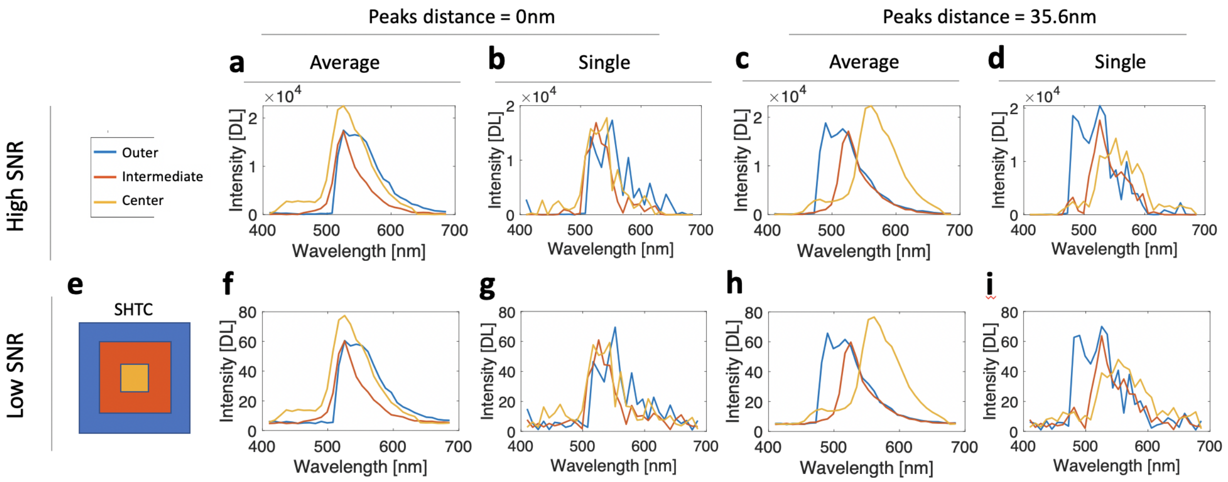

Supplementary Figure 22. Spectra of extreme conditions in SNR-Overlap simulation. The extremes of the simulation utilized in Supplementary Figure 20 are reported here as spectra for comparison. For high signal-to-noise ratio (**a**) average spectrum for spectra with peak-maxima distance set to zero and (**b**) example single spectra from each concentric square region of the simulation (digital levels, DL). (**c**) Average and (**d**) single spectra at high SNR for simulation with spectra separated with a peak-to-peak distance of 35.6nm. (e) Reference Simulated Hyperspectral Test Chart with color coded concentric squares. The low SNR simulation spectra are reported here for a peak distance of zero as (**f**) average and (**g**) single and for a peak distance of 35.6nm as (**h**) average and (**i**) single.

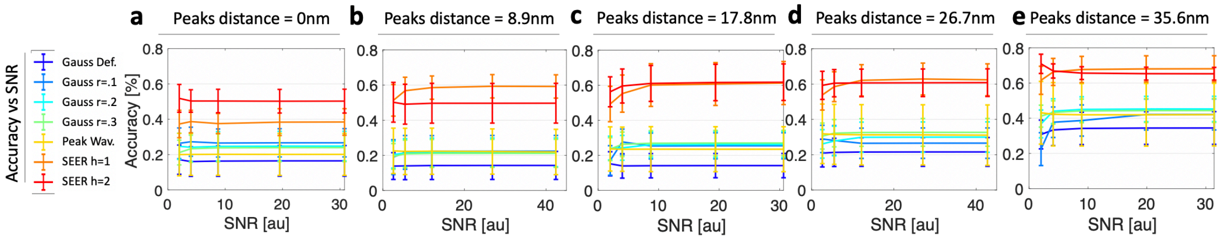

Supplementary Figure 23. Spectral separation accuracy of SEER under different spectral overlap and SNR conditions. Spectral separation accuracy was calculated for different signal-to-noise ratios and spectral maxima separation (**a**) aligned, (**b**) 8.9nm, (**c**) 17.8nm, (**d**) 26.7nm, (**e**) 35.6nm, starting from the visualizations in Supplementary Figure 20 and corresponding spectra in Supplementary Figure 21. The spectral separation accuracy is calculated here as the sum of the Euclidean distance of the RGB vectors between pairs of the concentric squares of the simulation, in ratio to the largest color separation (red to green, red to blue, blue to green). A thorough description of spectral separation accuracy calculation is reported in the Methods section. Each value in the plots represents the average distance of 200^2 pixels; error bars are the standard deviation of normalized spectral separation accuracy value across all pixels. The average spectral separation accuracies over multiple SNR conditions for each spectral maxima separation: (**a**) with highly overlapping spectra SEER provides on average 38.0% for harmonic 1, 50.6% for harmonic 2, while the best performing other comparison here is Gaussian r=.3 with an average 26.7%. (**b**) With a 8.9nm peak-to-peak separation SEER h=1 averages 57.0%, SEER h=2 49.6%, other best performing here is Peak Wavelength with 22.2%; (**c**) with 17.8nm separation SEER h=1 averages 57.2%, SEER h=2 60.0±2.3%, other best Gauss r=.3 with 26.2% (**d**) with 26.7nm separation SEER h=1 averages 59.9%, SEER h=2 60.4%, other best Gauss r=.3 with 32.1%; (**e**) with well separated spectra 35.6nm apart, SEER h=1 averages 66.3%, SEER h=2 66.7%, with other best Gauss r=.3 scoring 43.5% in average.

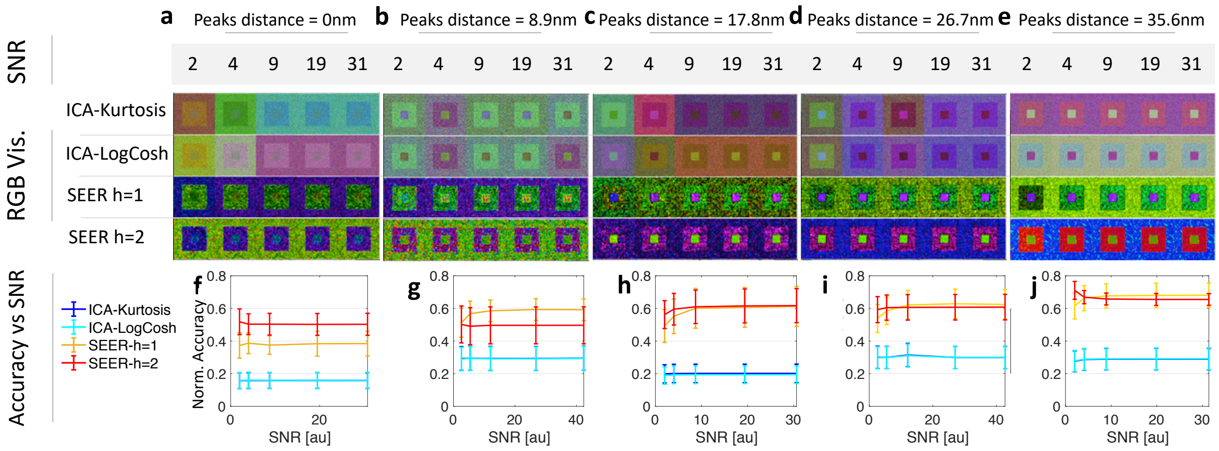

Supplementary Figure 24. Comparison of SEER and ICA spectral image visualization (RGB) under different spectral overlap and SNR conditions. The same simulation used in Supplementary Figure 20, which changes parameters for the Simulated Hyperspectral Test Chart obtaining different values of peak-to-peak spectral overlap and signal to noise, is used here to compute the spectral separation accuracy (Methods) for Independent Component Analysis using ENVI, with 3 independent components (ICs) with optimization for this specific dataset. (**a,b,c,d,e**). The three ICs are utilized as R, G, B channels for creating a color image for each simulation parameter (ICA = 3 line) and are shown here next to SEER harmonic 1 and 2 (SEER h=1 and SEER h=2 respectively). Error bars are the standard deviation. (**f,g,h,i,j**) The parameters of spectral separation accuracy described in the methods section are applied here to the SEER and ICA results. Each value in the plots represents the average distance of 200^2 pixels and error bars are the standard deviation of normalized spectral separation accuracy value across all pixels. Overall spectral separation accuracy of ICA, as calculated here, averages at 24.8% for Kurtosis function (ICA-K), 24.7% for LogCosh (ICA-L), while SEER's 55.7% h=1 and 57.5% h=2. For the different levels of overlaps the average spectral separation accuracy are (**f**) ICA-K 15.8%, ICA-L 15.9%, SEER 38.0% for harmonic 1, 50.6% for harmonic 2; (**g**) ICA-K 29.4%, ICA-L 29.2%, SEER h=1 57.0%, SEER h=2 49.6%; (**h**) ICA-K 20.1%, ICA-L 19.2%, SEER h=1 57.2%, SEER h=2 60.0%; (**i**) ICA-K 30.3%, ICA-L 30.1%, SEER h=1 59.9%, SEER h=2 60.4%; (**j**) ICA-K 28.6%, ICA-L 28.8%, SEER h=1 66.3%, SEER h=2 66.7%;

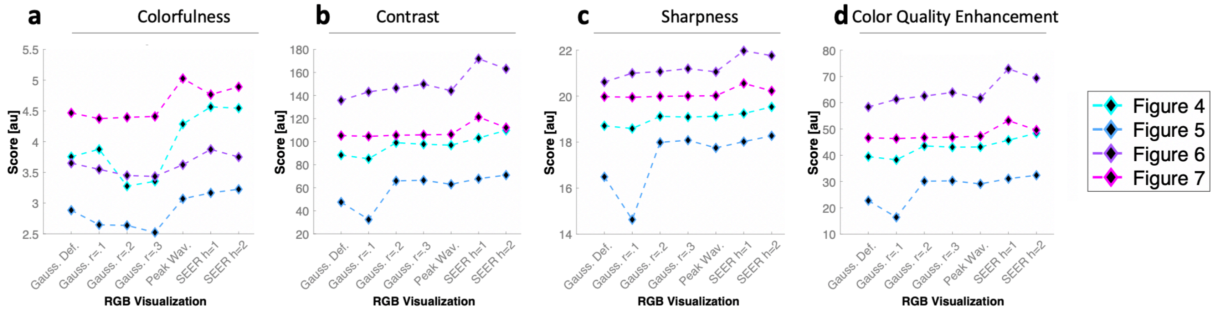

Supplementary Figure 25. Quantification of enhancement for Figures 4-7. The scores of (a) colorfulness, (b) contrast, (c) sharpness and (d) Color Quality Enhancement (CQE) are calculated according to Methods section for multiple visualization strategies. Average values are reported in Supplementary Table 2 and 3. (a) Colorfulness values for SEER were generally higher than other approaches, made exception for Figure 7 Peak Wavelength visualization (reported in Supplementary Figure 19d), owing to a very low average intensity in the red channel ($\langle I_R \rangle=840$), and an almost double average green to blue intensity ($\langle I_G \rangle / \langle I_B \rangle=1.7$), which makes the β parameter used in colorfulness small in average and the denominator of the second logarithm in the colorfulness equation (Methods) approximately equal to 1, producing a factor of 10 larger than usual ratio of variance β to average β. This combination of intensities results in a colorfulness 1.03-fold higher than the SEER h=2, however in this case the value of colorfulness does not correspond to human observation (Supplementary Figure 19d) suggesting this score could be an outlier due to a special combination of intensities. The values of (**b**) contrast, (**c**) Sharpness show higher performance for SEER. (**d**) The CQE score of SEER was higher than the standard, with improvement of 11%-26% for Figure 4, 7%-98% for Figure 5, 14%-25% for Figure 6 and 12%-15% for Figure 7.

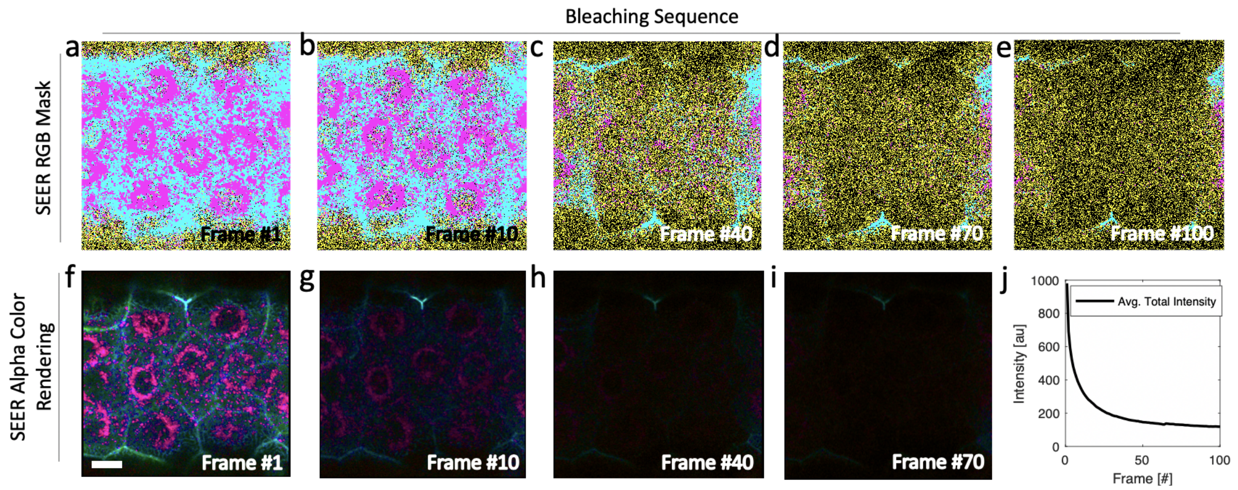

Supplementary Figure 26. Visualization of photobleaching with SEER. Photo-bleaching experiments were performed on a 24 hpf zebrafish embryo *Gt(cltca-citrine); Tg(fli1:mKO2); Tg(ubiq:memTdTomato)*, labeling clathrin, pan-endothelial and membrane respectively. The experiments were performed utilizing the "bleaching" modality in the Zeiss Zen 780 inverted confocal, where single z positions were acquired in lambda mode. Frames are acquired every 13.7 sec, with 5 intermediate bleaching frames (not acquired) at high laser power until image intensity reached 90% bleaching. The SEER RGB mask represents the values of colors associated to each pixel, independent from the intensity values. The map used here is Radial map in Center of Mass mode. In this modality the map will adjust its position on the shifting center of mass of the phasor clusters, visually compensating for the decrease in intensity**.** (**a**) In the initial frame the cltca-citrine is associated to a magenta color, membrane to cerulean, pan-endothelial is not in frame and background to yellow. (**b**) Frame 10 shows consistent colors with the initial bleaching; the colors are maintained (**c**) at frame 40 and (**d**) frame 70 where most of the signal has bleached and most colors have switched to yellow (here, background). (**e**) Final frame shows the 90% bleached sample. The Alpha Color rendering adds the information of intensity to the image visualization. Here we show for comparison (**f**) frame 1, (**g**) frame 10, (**h**) frame 40 and (**i**) frame 70. Scale bar 10μm. (j) Average total intensity plot as a function of frame, calculated from the sum of 32 channels, shows evident bleaching in the sample. Further visualization is provided in Supplementary Movie 3

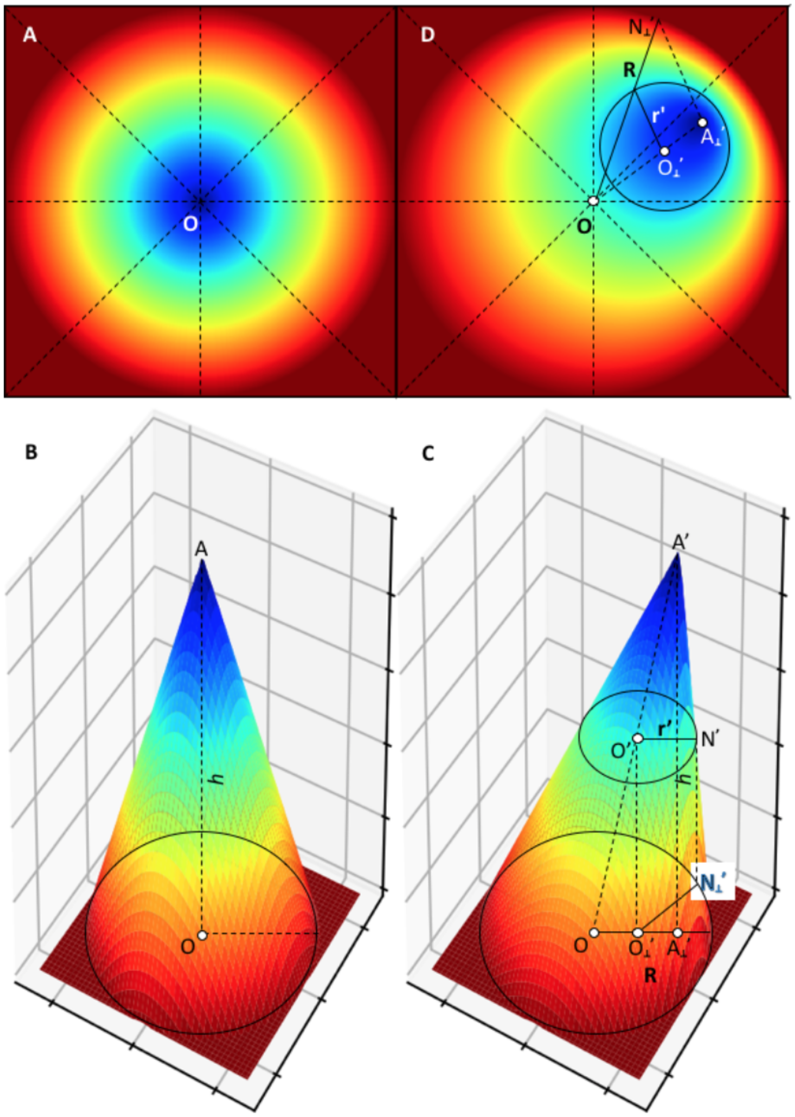

Supplementary Figure 27. Morph mode algorithm pictorial abstraction. (**a**) A Radial map in standard mode centered at the origin O can be abstracted as a (**b**) 3D Conic shape with height h and apex A. (**c**) Upon shifting the apex of the cone from A to A', the map reference center translates from origin O to the projection $A_{\perp}'$. During this shift, the edges of the cone base are anchored on the phasor unit circle. (**c-d**) If we consider a plane cutting the oblique cone horizontally, the resulting section is a circle with center O' and radius r'. The projection of this circle is centered on $O_{\perp}'$ which lies on the line $OA_{\perp}'$, adjoining the fixed center O and new apex projection $A_{\perp}'$ and has the same radius r'. As a result, (**d**) all of the points in each of these projected circles are shifted along the vector $OO_{\perp}'$ on the phasor plot.

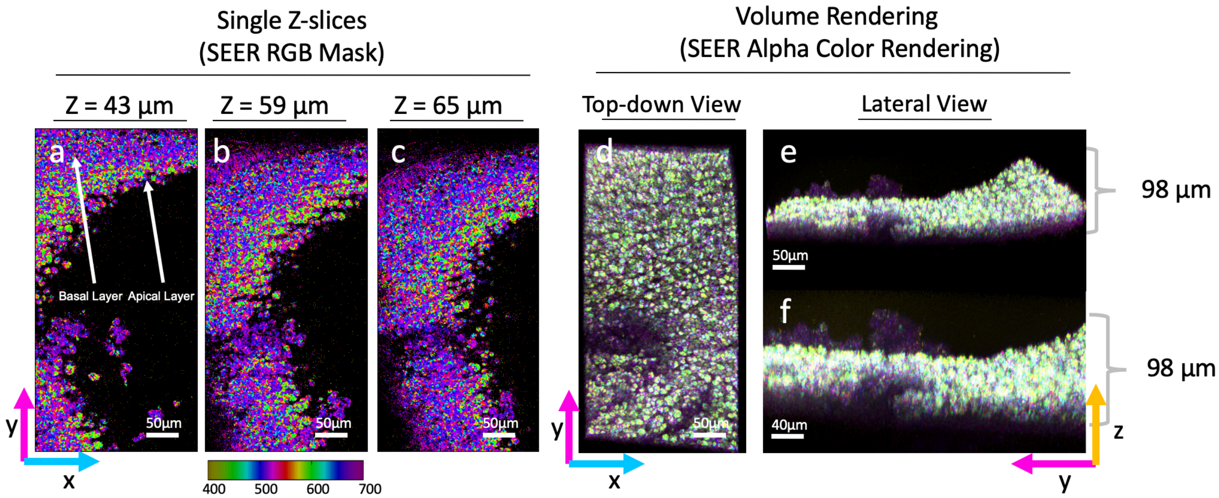

Supplementary Figure 28. Autofluorescence visualization in volumetric data of unlabeled freshly isolated mouse tracheal explant. A tiled z-stack (x,y,z) imaged with multi-spectral two-photon microscopy (740nm excitation, 32 wavelength bins, 8.9nm bandwidth, 410-695 nm detection) is here visualized as a single (x,y) z-slice SEER RGB Gradient Descent Max Morph mask at (**a**) 43µm, (**b**) 59µm, (**c**) 65µm depth. Color differences between basal and apical layer cells are maintained at different depths, with consistent hue for each of the cell layers. Colorbar represents the main wavelength associated to one color in nanometers. Volume renderings presented as SEER Alpha Color renderings for (**d**) top-down (x,y) view, (**e**) Lateral (y,z) view and (**f**) zoomed-in lateral (y,z) view show the shape and the 98µm thickness of the unlabeled tissue sample.

Supplementary Note 1
Choice of harmonic for visualization

The distribution of spectral wavelengths on the phasor plot is highly dependent on the harmonic number used. Typically, the first and second harmonics are utilized for obtaining the hyperspectral phasor values due to visualization limitations imposed by branching within the Riemann surfaces in complex space[1].

The first harmonic results in a spectral distribution which approximately covers $3/2\pi$ radians for spectrum within the visible range (400 nm – 700 nm), within the universal circle, along a counterclockwise path. As a result, spectra separated by any peak-to-peak distance will appear in different positions on the phasor plot. However, the first harmonic provides a less efficient use of the phasor space, leaving $1/2\pi$ radians non utilized and leading to a lower dynamic range of separation as can be seen in Supplementary Figure 9 and 10.

Similarly, the second harmonic approximately spans over (3/2 + 2) radians on the phasor for spectrum within the visible range (400 nm – 700 nm), distributing spectra in a more expansive fashion within the universal circle, simplifying the distinction of spectra which may be closely overlapping and providing a higher dynamic range of separation as demonstrated in Supplementary Figure 6 and 7. The downside of this harmonic is the presence of an overlap region from orange to deep red fluorescence. Within this region, spectra separated by 140 nm (in our system with 32 bands, 410.5 nm to 694.9 nm with 8.9 nm bandwidth), may end up overlapping within the phasor plot. In this scenario, it would not be possible to differentiate those well-separated spectra using the second harmonic, requiring the use of the first. Thanks to SEER, the choice of which harmonic to use for visualization can be quickly verified and changed within the HySP software (http://bioimaging.usc.edu/software.html).

In the common scenario of imaging with a single laser line, the range of the majority of the signal emitted from multiple common fluorophores is likely to be much smaller than 150 nm due to the Stokes' shift which is usually in the 20-25 nm range. Excitation spectra of fluorescent proteins separated by 140nm generally do not overlap, requiring utilization of a second excitation wavelength to obtain the signal.

The SEER method presented here has utilized the second harmonic in order to maximize the dynamic range of the phasor space and separate closely overlapping spectra. However, SEER can work with the first harmonic seamlessly, maintaining swift visualization of multiple fluorophores that may be far in peak spectral wavelength.

Supplementary Note 2
Color visualization limitations for SEER

The SEER maps are built based on the frequency domain values generated by applying the phasor method to hyperspectral and multispectral fluorescent data. RGB colors are used to directly represent these values. As such, the quality of color separation has a maximum resolution limited by the spectral separation provided by the phasor method itself. Therefore, as long as the phasor method can differentiate between spectra which have a higher amount of fluorescent signal vs noise (high signal to noise) and spectra with a higher combination of the noise versus the signal (low signal to noise), the SEER maps will assign different colors to these spectra. If signal is indistinguishable from noise, SEER maps will assign the same color to these spectra. In the scenario where spectra derived from two different effects are exactly the same, for example, a case where low protein expression is on an outer layer and a high level expression is attenuated at a deeper level, the phasor method and the SEER maps, in their current implementation, will not be able to differentiate between the two effects. The separation of these two effects is a different and complex problem which depends on the optical microscopy components, the sample, the labels, the multispectral imaging approach and more factors in the experimental design, and we believe this separation falls outside of the scope of this paper and constitutes a project on its own.

Supplementary Note 3

Measuring color contrast in fluorescent images

There is an inherent difficulty in determining an objective method to measure the image quality of color images in relation to fluorescent images. The main challenge for fluorescent images is that for the majority of fluorescence microscopy experiments, a reference image does not exist because there is an inherent uncertainty related to the image acquisition. Therefore, any kind of color image quality assessment will need to be based solely on the distribution of colors within an image.

This type of assessment has its own further challenges. Although there have been a variety of quantitative methods formulated to determine the quality of intensity distributions in grayscale images, such methods for color images are still being debated and tested[9-12]. This lack of suitable methods for color images mainly comes from the divide between the mathematical representation of the composition of different colors and human perception of those same colors. This divide occurs because human color perception varies widely and is nonlinear for different colors; whereas the quantitative representation of any color is usually a linear combination of base colors such as Red, Green, and Blue. This nonlinear human perception of color is closely related to the concept of hue. Loosely speaking, hue is the dominant wavelength that reflects the light. Hues perceived as blue tend to reflect light at the left end of the spectrum and red at the right end of the spectrum. Generally, each individual color has a unique holistic trait which is determined by its distinctive spectrum. Discretization of the spectrum into multiple components cannot fully describe the original richness in color.

The current methods for determining the quality of an RGB image usually adapt grayscale methods in two different ways. The first method involves either converting the three channel color image into a single channel grayscale image before measuring the quality. The second method measures the quality of each channel individually and then combines those measurements with different weights. However, both methods face limitations in providing a value of quality that correlates well with human perception. The first method loses information when converting the color image to grayscale. The second method tries to interpret the nonlinear human perception of the quality of a color image by separating it into three channels and measuring them individually. The inherent hue of a color, however, is more than the sum of the individual component colors, since each channel taken individually is not necessarily as colorful as the combined color.

A more complete color metric should take hue into account, such as by measuring the colorfulness loss between the original and processed images[10]. In conclusion, as a consequence of this limitation in measuring colorfulness for current methods, there is currently no established "true measure of contrast" within fluorescent color images.

References:

1. Cutrale, F. *et al.* Hyperspectral phasor analysis enables multiplexed 5D in vivo imaging. *Nat. Methods* **14,** 149–152 (2017).
2. Browne, A. W. *et al.* Structural and functional characterization of human stem-cell-derived retinal organoids by live imaging. *Investig. Ophthalmol. Vis. Sci.* (2017). doi:10.1167/iovs.16-20796
3. Stringari, C. *et al.* Phasor approach to fluorescence lifetime microscopy distinguishes different metabolic states of germ cells in a live tissue. *Proc. Natl. Acad. Sci.* **108,** 13582–13587 (2011).
4. Sharick, J. T. *et al.* Protein-bound NAD(P)H Lifetime is Sensitive to Multiple Fates of Glucose Carbon. *Sci. Rep.* (2018). doi:10.1038/s41598-018-23691-x
5. Ranjit, S., Malacrida, L., Jameson, D. M. & Gratton, E. Fit-free analysis of fluorescence lifetime imaging data using the phasor approach. *Nat. Protoc.* **13,** 1979–2004 (2018).
6. Lakowicz, J. R., Szmacinski, H., Nowaczyk, K. & Johnson, M. L. Fluorescence lifetime imaging of free and protein-bound NADH. *Proc. Natl. Acad. Sci.* (1992). doi:10.1073/pnas.89.4.1271
7. Stringari, C. *et al.* Multicolor two-photon imaging of endogenous fluorophores in living tissues by wavelength mixing. *Sci. Rep.* (2017). doi:10.1038/s41598-017-03359-8
8. Skala, M. C. *et al.* In vivo multiphoton microscopy of NADH and FAD redox states, fluorescence lifetimes, and cellular morphology in precancerous epithelia. *Proc. Natl. Acad. Sci.* (2007). doi:10.1073/pnas.0708425104
9. Panetta, K., Gao, C. & Agaian, S. No reference color image contrast and quality measures. IEEE Trans. Consum. Electron. (2013). doi:10.1109/TCE.2013.6626251
10. Hasler, D. & Suesstrunk, S. E. Measuring colorfulness in natural images. in *Human Vision and Electronic Imaging VIII* (2003). doi:10.1117/12.477378
11. Sos S. Agaian and Karen P. Lentz and Artyom M. Grigoryan. A New Measure of Image Enhancement. IASTED Int. Conf. Signal Process. \& Commun. (2000).
12. Agaian, S. S., Silver, B. & Panetta, K. A. Transform coefficient histogram-based image enhancement algorithms using contrast entropy. IEEE Trans. Image Process. (2007). doi:10.1109/TIP.2006.888338
