# Supplementary material for: Pre-processing visualization of hyperspectral fluorescent data with Spectrally Encoded Enhanced Representations
Source: Nat Commun. 2020 Feb 5;11:726. doi: 10.1038/s41467-020-14486-8 (PMC7002680; doi:10.1038/s41467-020-14486-8)
Supplement: Supplementary file 3 — Description of Additional Supplementary Files [file 41467_2020_14486_MOESM3_ESM.pdf]

Description of Additional Supplementary Files

File name: Supplementary Movie 1
Description: Maximum intensity projection rendering of a single fluorescence label against multiple autofluorescences. *Tg(fli1:mKO2)* (pan-endothelial fluorescent protein label), here visualized using SEER angular reference map, clearly represents with different colors the contribute from fli1:mKO2 from that of autofluorescence from pigments and from yolk.

File name: Supplementary Movie 2
Description: Maximum intensity projection rendering of a triple label fluorescence zebrafish embryo *Tg(kdrl:eGFP); Gt(desmin-Citrine);Tg(ubiq:H2B-Cerulean)* labelling respectively vasculature, muscle and nuclei. Here TrueColor 32 channel visualization is compared with SEER angular map in center of mass mode and with SEER gradient descent map in max morph. Phasor plots with corresponding color coding are reported.

File name: Supplementary Movie 3
Description: Maximum intensity projection rendering of photo-bleaching experiment on a zebrafish embryo *Gt(cltca-citrine); Tg(fli1:mKO2); Tg(ubiq:memTdTomato)*, labeling clathrin, pan-endothelial and membrane respectively. SEER angular map in center of mass mode was here utilized for visually compensating the effects of photo-bleaching in the sample. SEER RGB mask associates constant colors to the different fluorescence signals, until signal-to-noise renders them undistinguishable from background. Alpha color visualization, which accounts for intensity values in pixels, shows a progressive disappearance of the signal until 90% bleaching is achieved.

File name: Supplementary Movie 4
Description: 3D plot visualization of the shifted-cone approach utilized in morph mode.
